# Assessing Actual Strategic Behavior to Construct a Measure of Strategic Ability

**DOI:** 10.3389/fpsyg.2018.02750

**Published:** 2019-01-18

**Authors:** Ennio Bilancini, Leonardo Boncinelli, Alan Mattiassi

**Affiliations:** ^1^IMT School for Advanced Studies Lucca, Lucca, Italy; ^2^Dipartimento di Scienze per l'Economia e l'Impresa, Università degli Studi di Firenze, Firenze, Italy; ^3^Dipartimento di Economia “Marco Biagi”, Università degli Studi di Modena e Reggio Emilia, Modena, Italy

**Keywords:** strategic thinking, rationality, mentalization, cognitive skills, game theory, depth of reasoning, strategic success

## Abstract

Strategic interactions have been studied extensively in the area of judgment and decision-making. However, so far no specific measure of a decision-maker's ability to be successful in strategic interactions has been proposed and tested. Our contribution is the development of a measure of strategic ability that borrows from both game theory and psychology. Such measure is aimed at providing an estimation of the likelihood of success in many social activities that involve strategic interaction among multiple decision-makers. To construct a reliable measure of strategic ability, that we propose to call “Strategic Quotient” (SQ), we designed a test where each item is a game and where, therefore, the individual obtained score depends on the distribution of choices of other decision-makers taking the test. The test is designed to provide information on the abilities related to two dimensions, mentalization and rationality, that we argue are crucial to strategic success, with each dimension being characterized by two main factors. Principal component analysis on preliminary data shows that indeed four factors (two for rationality, two for mentalization) account for strategic success in most of the strategically simpler games of the test. Moreover, two more strategically sophisticated games are inserted in the test and are used to investigate if and to what extent the four factors obtained by simpler games can predict strategic success in more sophisticated strategic interactions. Overall, the collected empirical evidence points to the possibility of building a SQ measure using only simple games designed to capture information about the four identified factors.

## 1. Introduction

Human life in society is pervaded with strategic interactions, namely situations where at least two decision-makers interact with each other to obtain prizes that are scarce, and that are assigned depending on the result of the interaction itself: all decision-makers take an action among a set of feasible actions in order to achieve the goal (winning or ranking better than others), and the results of competition do not depend only on the action taken by a single player, but on the whole profile of actions taken by all players. In human society, many activities are based on competition, such as those involved in markets, sports, politics (even marriage can be seen as competition for a partner).

Strategic behavior has been formally modeled by game theory (Morgenstern and Von Neumann, 1944), typically postulating perfect rationality of players (i.e., participants to a strategic interaction), meaning that they always take the best actions to obtain their goals, and that they have consistent expectations on others' behaviors (Mailath, 1998). More recently, experimental economics has provided evidence for large discrepancies between actual data and game-theoretic predictions with rational players (Samuelson, 2005; DellaVigna, 2009). In particular, the assumption that individuals have an abundance of cognitive resources has been questioned, and the implications of its removal analyzed (Simon, 1955, 1972)^1^. Thus, novel mathematical models of behavior have been developed in order to account for experimental results, giving rise to behavioral game theory (Camerer, 2003). Indeed, departure from perfect rationality can be modeled by assuming that individuals are prone to mistakes when making decisions: some mistakes are systematic and depend on the heuristics followed as rules of behavior (Kahneman, 2003), others are stochastic and result from lack of control on the decision process (Young, 2001; see Javarone and Atzeni, 2015 for a recent application). Another relevant issue concerns whether rationality is aimed at pursuing individual self-interest (*individual rationality*) or, rather, it is directed to overcome potential conflicts of interest and to reach goals at the group level (*collective rationality*, see Kirman, 2010, for a critical discussion).

Within economics and game theory, the only attempt to measure individual strategic skills has focused on strategic sophistication as the ability of individuals to compute recursive best response (see section 3.6). Conversely, psychologists have long provided tools for measuring empirically many abilities of individuals. One of the most influential and widely adopted constructs is the Intelligent Quotient (IQ hereon), that was developed to easily predict the general human functioning. It is usually calculated on several subtests results (see for example Wechsler, 2008), each one measuring specific cognitive competences (such as short-term memory) or a specific cognitive domain (such as mathematical reasoning). However, IQ tests are generally useful in predicting school and academic success (not coincidentally, both IQ and school or graduation final mark heavily depend on the average of other measures of vastly different—and partly independent—skills) or for identifying individuals who depart drastically from population average, but lack in several areas (Stanovich, 2009), such as capturing the difference between judgment and decision-making^2^. The mere existence of such discrepancy undermines our knowledge of human mental processes in a strategic context and asks for a dedicated measure of cognitive skills in strategic environments.

Our contribution is to borrow from both game theory and psychology, in particular psychometrics, in order to propose a measure of cognitive skills that is useful to predict success in competitive environments. We point out that, differently from traditional psychometric tests in which individual attributes are compared with normative data, here we cannot score a test in isolation from others, since in a strategic environment what is optimal for an individual must depend on the behaviors of other players.

In section 2 we first present the theoretical background, and we then introduce and discuss the proposed test. In section 3 we show our results, extracting factors by means of the principal component analysis (PCA hereon), and providing synthetic measures of strategic abilities, namely SQ8 and SQfactor; finally, we test the predictive power of the identified factors and synthetic measures, and we explore their relation with other economic, educational and psychological variables, and with models of strategic sophistication. In section 4 we briefly comment on our findings, and we sketch directions for future developments.

## 2. Materials and Methods

### 2.1. Theoretical Framework

With the term *rationality* we refer to the cognitive abilities of a player that subtend reasoning, monitoring, planning actions, setting, and reaching goals, which are likely to help strategic success (Gill and Prowse, 2016). Rationality roughly corresponds to the so-called “executive functions” (Jurado and Rosselli, 2007; Diamond, 2013), which are known to be developmentally stable (Miyake and Friedman, 2012) and are assessed in clinical neuropsychological settings by using tasks that tap differently in one function or another (Miyake et al., 2000). Individual differences in executive functions are known to affect decision making (Del Missier et al., 2010, 2012). One of the core executive functions, inhibitory control, allows the individual to override her or his impulse toward a lure in order to act in the most appropriate goal-related way. Inhibitory control might play a fundamental role in developing strategies that avoid taking the route to the best possible outcome when this would ultimately result in a suboptimal payoff given the structure of the interaction (Alós-Ferrer et al., 2016b; Alós-Ferrer, 2018).

***Example 1.*** Say for example that an interaction is structured as follows: a player, termed “proposer,” chooses two out of three possible outcomes (A, B, and C), then another player, termed “responder,” chooses one out of the two that were chosen by the proposer; both players get the outcome chosen by the responder. Each player has personal preferences over A, B, and C: the proposer prefers A to B and B to C (as such, s/he also prefers A to C), while the responder prefers B to A and A to C (as such, s/he also prefers B to C). The intuitive strategy for the proposer would be to choose the preferred outcomes for her/himself, i.e., A and B. However, by taking into account the preferences of the receiver (s/he prefers B to A), the proposer would come to the conclusion that the final payoff for that choice would be the suboptimal B outcome. If s/he wants the best payoff for her/himself among the three, the pair that s/he needs to propose is not A and B, but A and C, since the responder prefers A to C.

In a strategic setting, it seems reasonable to propose a distinction within rationality between *optimization*—i.e., the ability to identify the optimal course of action to achieve the preferred goal in a fixed environment—and *iteration*—i.e., the ability to take into consideration that the environment is not given, but determined by other players' choices, which are presumably taken to achieve their own goals. In the previous example, iteration was present at a very simple level, just involving one step of—as it is called in game theory—backward reasoning. However, iteration may be much more demanding, as it is illustrated in Example 2 (which is also part of our test to measure strategic skills).

***Example 2.*** In this game, called *p*-Beauty Contest (Nagel, 1995), a number of players try to guess a number that is the closest to a fraction *p* of the mean of others' guesses. The player that guesses the closest number wins the game (in case of a draw, all drawing players win). Let's say that the range from which a player can guess is 0–90 and *p* = 2/3. Assuming that all other players choose a number at random, a player should guess 30 as the number most close to the 2/3rds of the mean choice of others (being 45 if choices are indeed random). However, if one assumes the others to behave like the player above mentioned, the new best guess would be 20 (as the closest number to the 2/3rds of 30). In turn, the others may behave like this latter player, and so on. As it clearly appears, higher order iteration pushes the (theoretically) optimal guess toward 1 (note that 1 is the best guess when everybody else guesses 1).

Example 2 shows that iteration can be very long and articulated in strategic settings. Not only, it also opens to a following question: how long should actually be iteration to imply success? The answer clearly depends on who are the other players. Indeed, the literature on beauty contests highlights that players typically guess a number far away from 1 (Coricelli and Nagel, 2009). Hence, in addition to rationality, one's own knowledge about others is likely to play an important role in determining success in strategic settings.

The knowledge about others is a complex domain that is poorly understood (Krauss and Fussell, 1991). It is clear that being able to correctly evaluate others' behavior requires skills that are not merely information-driven nor insight-driven, but also requires a number of mental processes that encompass specific evaluations and comparisons (Fe and Gill, 2018). Knowledge about others is known to be based on one's own knowledge (Epley et al., 2004; Mitchell et al., 2005, 2006) and biased toward it (Nickerson et al., 1987; Dunning and Cohen, 1992; Nelson et al., 1998). Furthermore, others' knowledge-related factors are known to affect strategic decisions (see for example Fabre et al., 2016, and Cubel and Sanchez-Pages, 2017, on gender stereotypes). Following (Nickerson, 1999), “knowledge about others” can be seen as the pool of beliefs, opinions, assumptions and attitudes, and the states of mind that relate to them. According to the *theory of mind* (see for example Premack and Woodruff, 1978; Stone and Gerrans, 2006), attributing mental states allows people to understand that the information processed in others' minds might be different from one's own mind, and to predict intentionality. In the following, we will refer to the ability to form correct beliefs about others' aims and skills as *mentalization*. One of the most important aspects of the knowledge of others is their cognitive skills. Example 2 shows how a player able to compute iteration might make different decision if predicting other players to be more or less able to compute iteration (guessing 1 in the first case or a number away from 1 in the second).

Cognitive skills represent an important aspect of the knowledge of others, but is not the only one. Among many, we focus on others' preferences. Therefore, we propose a distinction within mentalization between others' *preferences*—i.e., the goals that other players are trying to reach—and *skills*—i.e., the cognitive abilities that other players employ for reaching their goals. Example 3 illustrates the relevance of knowing others' preferences.

***Example 3.*** A seller aims at selling a good/service to a pool of buyers, with no knowledge about their willingness to pay. In order to maximize profits, the seller has to properly take into account the reduction in the quantity sold due to an increase in price. This is likely to be based, mainly, on rationality. Before than that, however, the seller should correctly assess buyers' willingness to pay, which is something related to other players' preferences.

In sum, we propose that strategic thinking might be influenced by the rationality of the individual, covering both the ability to identify the optimal course of action to achieve a goal in a given environment—called optimization—and the ability to take into consideration that the environment is determined by other players' choices—termed iteration, and the mentalization component, covering the abilities to mentally represent both others' preferences and others' skills. While generally rationality allows individuals to grasp the structure of the game and its payoffs, mentalization allows players to predict other players' behavior.

### 2.2. Measurement of Cognitive Abilities

Traditional methods for IQ estimation (e.g., Wechsler, 2008) consist in a timed test which includes a range of problems to solve. A score is assigned on the basis of the answers given by test takers and the score is then converted into an IQ value according to appropriate criteria and scale. Our ultimate aim is to provide a measure of strategic abilities that uses the same method of calculation as the IQ, but that differs in terms of the dimension (i.e., construct) to be measured. In particular, we are not interested to measure cognitive skills in general, rather we focus on the particular abilities which are most relevant to the problem of making the best decision in a strategic contest, namely in a situation in which the outcome of one's own decisions depends critically not only on own choices but also on the choices of others. In scientific studies on interactions between individuals, the term “game” is used to indicate a stylized strategic situation—a circumscribed streamlined model with precise rules—of strategic situations which may be encountered in the real world. Wars, Olympic competitions, courting practices, commercial disputes, well drilling plans searching for hydrocarbon, and negotiations for agreements regarding pollution reduction are all strategic situations. Most of them are competitive in nature, meaning that the success of one implies the failure of some other. All these are formally representable as games, in a game-theoretic sense.

With this aim, we have elaborated a list of games—some of which are commonly studied in the game-theoretic literature—presumably involving the use of those skills that are crucial to success in typical strategic settings. The procedure that we have followed to administer the test to participants, and a summary description of its content, is provided here in the following, while more specific details can be found in the Appendix and Supplementary Material.

#### 2.2.1. General Data

Participants were asked to provide quite accurate general data, with particular emphasis on education and socio-economic condition. The time used to fill these data was in between 5 and 10 min.

#### 2.2.2. Pre-test

Participants were administered (see p. 1 of the Supplementary Material) a few preliminary questions in order to assess their risk-preference, time-preference, and social preferences as recipient in an ultimatum game. Furthermore, they had to answer three questions concerning pattern recognition in numerical and graphical sequences, and propositional logic. The last question of this block was about their willingness to pay for a reliable measure of their strategic ability. No time constraint was imposed on this task, with an average time of completion being around 5 min (and no one using more than 10 min). We explicitly told participants that these questions were not used to measure strategic skills, but the aggregate pattern of their answers would have been objects to following questions.

#### 2.2.3. Instructions

Participants were given instructions (see p. 2 of the Supplementary Material). They were invited to read them with care and to ask questions in case of any doubt. Instructions stressed that optimal choices depend on the choices of all participants to the test. Three to 5 min were generally enough to complete this phase.

#### 2.2.4. Test

Participants were administered the test, comprising various questions organized in ten games (p. 3 to 7 of the Supplementary Material). Participants were told that they should complete the test in about 30 min, but were flexible in giving them five more minutes if required. In the following we provide a concise description of all 10 games.

Game 1 asks about one's own belief about the distribution of answers to pre-test questions. Game 2 asks about one's own belief about the distribution of beliefs about answers to pre-test questions. Game 3 asks to take choices in two different roles that interact together: in one role (labeled *Tizio*) the optimal choice is given, while in the other role (labeled *Caio*) the optimal choice depends on Tizio's choice, which in one case is given by the distribution of behaviors when in the other role, while in another case is taken by a perfectly task-maximizing automaton. Game 4 describes an interaction between two roles (labeled again as Tizio and Caio) framed as weapon shooting: Tizio has to choose among different weapons, and then Caio has to choose which part of Tizio's body to aim at when shooting; participants are informed of probability of shooting successfully for each combination of weapon and body part. This game asks first to make a decision as Tizio, and then to make a decision as Caio, supposing in one case that Tizio's behavior is given by the distribution of choices by other participants to the test, and in another case by an automaton. Game 5 asks to choose the color and the number, among given alternatives, that are predicted to be mostly chosen by other participants to the test. While for the choice of color no additional information is given, for the choice of the number a implicit suggestion toward a specific number is given (acting as a potential focal point, see Schelling, 1980). Game 6 (typically known as *beauty contest*, see Nagel, 1995) asks to choose a number in between 1 and 90 that is the closest to 2/3 of the average of others' choices, where “others” are all participants to the test in one case, only participants who provided correct answers to all pre-test questions on pattern recognition and propositional logic in another case, and a task-maximizing automaton in a third case. Game 7 (typically known as *ultimatum game*, see Güth et al., 1982) presents a dilemma where one role (Tizio) has to choose how to split one hundred euros between herself and another role (Caio), with the latter having then to choose whether to accept the proposed split, making it effective, or to reject it, with the consequence that both roles obtain zero euros; participants have to take decisions in the role of Tizio only, assuming that in one case Caio's behavior is given by the distribution of choices in the pre-test question when assessing social preferences as recipient in an ultimatum game, while in the other case it is given by the usual automaton. Game 8 asks to give a selling price for a test which reliably measures strategic skills; the objective here is to maximize revenues, assuming that buying decisions are determined by the declarations about willingness to pay for such a test provided in the pre-test.

In Game 9 a scenario is presented where there are two armies confronting each other, and whose commanders are the usual Tizio and Caio; Tizio has to choose whether to burn all his bridges, which would prevent any possibility of retreat; after this choice is taken, and is observed by Caio as well (this allows for implicit communication of intentions based on *forward induction*, i.e., inferring intentions under the belief that players always behave rationally even when acting unexpectedly (see Govindan and Wilson, 2009), then Tizio and Caio have to choose simultaneously whether to attack or retreat. Here participants have to choose how to behave in the roles of Tizio and Caio, considering actual behaviors by other participants to the test; in one case, Caio's choice is given by looking only at those who provided correct answers to all pre-test questions on pattern recognition and propositional logic. In Game 10 (which can be seen as a simple version of the *centipede game*, see Rosenthal, 1981) a scenario is presented where two players, again Tizio and Caio, have the possibility to make a decision at different stages of the game in a sequential way (Tizio moves first); each time a player is called to make a decision, he has to choose whether to terminate the game and take a slightly larger fraction of a given stake for himself, leaving the remaining fraction to the other player, or to continue the game and give the chance to make a decision to the other player, and by doing so also increase the stake that will be shared. Similarly to previous games, the distribution of choices by participants to the test determine the relevant behavior of the other player for calculating actual scores; in addition, Tizio plays in one case against an automaton that that only cares about its own stake.

#### 2.2.5. Other Psychological Tests

Besides our test designed to measure strategic abilities, we administered to the same sample of subjects (after the completion of the main test) a number of other psychological tests: the BIG5 personality traits (Digman, 1990) with the 46-item inventory (it took approximately between 5 and 10 min to be completed) aimed at assessing the constellation of traits defined by the Five Factor Theory of Personality, the Raven's Advanced Progressive Matrices test with no age correction (APM, Raven and Court, 1998; it took approximately between 25 and 35 min to be completed) aimed at providing a measure of IQ, the Rational-Experiential Inventory with 40 items (REI40, Pacini and Epstein, 1999; it took approximately between 5 and 10 min to be completed) aimed at assessing the extent of rational and experiential information processing styles, and the Cognitive Reflection Test (CRT, Frederick, 2005) with 6 item (Primi et al., 2016; it took approximately between 3 and 6 min to be completed; see Alós-Ferrer et al., 2016a, 2018 for interpretation of this in terms of decision biases; see Stagnaro et al., 2018 for a test of CRT reliability) aimed at assessing individuals' ability to suppress an intuitive and spontaneous wrong answer in favor of a reflective and deliberate one.

### 2.3. Definition of Score Variables

Our use of score variables is theory-driven. We have used answers to Games from 1 to 8 to calculate game score variables and we have allocated them to four distinct groups, one for each dimension of rationality and mentalization. Scores for each part of Game 9 and Game 10 were kept separated and used to test the explanatory power of the factors extracted from the four mentioned groups of variables. The reason for this distinction is that the first eight games are relatively simpler, and each of them mainly requires the activation of one specific dimension of strategic ability. The last two games are instead more sophisticated and are likely to involve all dimensions of strategic ability to a non-negligible extent.

First, we have translated answers into numeric expressions measuring how successful the answer is (more successful answers receive higher points). Then, we have aggregated the scores of each participant within each game, if they were supposed to be driven by the same dimension of strategic ability, while we kept them separated otherwise. Finally, we have assigned each aggregated score to either rationality, distinguishing between optimization and iteration, or to mentalization, distinguishing between understanding of others' preferences and understanding of others' skills. Here in the following we provide the motivation behind our imputation procedure, while employed formulas can be found in Appendix A. In our notation, if we aggregate answers in a variable *gnXy* we mean that such answers come from game *n* (with *n* being a number between 1 and 10), success is mainly driven by dimension *X* (with *X* being either *R*, i.e., rationality, or *M*, i.e., mentalization), with *y* being its main component involved (where *y* is either *pref*, i.e., understanding of others' preferences, *skil*, i.e., understanding of others' skills, *opti*, i.e., ability to optimize, or *iter*, i.e., ability to iterate reasoning).

Scores for Game 1 and Game 2 concerning risk-preference, time-preference, social preferences and willingness to pay have been aggregated in *g*1*Mpref* and *g*2*Mpref* respectively, while the scores concerning how well other participants performed in pattern recognition and propositional logic have been aggregated in *g*1*Mskil* and *g*2*Mskil* respectively. Scores for Game 3 have been aggregated in *g*3*Ropti*, because even when optimal behavior depends on others' actual choices (when Caio has to match Tizio's choice, as resulting by the choices of other participants to the experiment), the best choice by Tizio is self-evident and hence knowledge of others' cognitive skill is supposed to play a negligible role. In Game 4, while scores as Caio are aggregated into *g*4*Ropti*, the two scores as Tizio are aggregated in *g*4*Riter*, since the actual (in one case) and optimal (in the other case) behavior by Caio, which can be quite articulated, should be appropriately taken into consideration, relying on iterated reasoning to properly determine what is own optimal behavior. The two scores for Game 5 both depend on coordination success, but while the one about colors is likely to be based only on the understanding of others' preferences, and hence is assigned to *g*5*Mpref*, the one about numbers has an evident anchor that can be used as a coordination device and its actual relevance is likely to depend on the understanding of others' skills, so that is assigned to *g*5*Mskil*. The success in Game 6 mainly depends on the ability of responders to best reply to others' (either participants' or automaton's) optimal behavior, hence relying on iterated reasoning, which is why we aggregated all scores in *g*6*Riter*. A similar reasoning holds for Game 7, leading us to aggregate all scores in *g*7*Riter*. Finally, the success in Game 8 mainly relies on the ability to understand how profits change as compound result of decreased demand and increased price, therefore we coded it as *g*8*Ropti*.

Finally, We calculated four distinct scores for Game 9—labeled from *g*91 to *g*94—and three distinct scores for Game 10 – labeled from *g*101 to *g*103, with the purpose of checking to what extent the information extracted from previous games can help to predict strategic success. Basically, we each of these scores codes whether the participant was successful in a single strategic decision.

Table 1 lists the games together with the dimensions, and their components, which should be mainly involved on the basis of the theoretical framework, along with the game type and the generated score variables.

**Table 1:** List of games with associated game types, main driver of strategic success, and generated score variables, as resulting from our theoretical framework.

**Game no**.	**Game type**	**Main skill**	**Score variable**
Game 1	Guess others' preferences	Mentalization–preferences	g1Mpref
	Guess others' skills	Mentalization–skills	g1Mskil
Game 2	Guess others' guesses	Mentalization–preferences	g2Mpref
	Guess others' guesses	Mentalization–skills	g2Mskil
Game 3	Trivial backward induction	Rationality–optimization	g3Ropti
Game 4	Backward induction	Rationality–optimization	g4Ropti
Game 5	Pure coordination	Mentalization–preferences	g5Mpref
	Pure coordination	Mentalization–skills	g5Mskil
Game 6	2/3 beauty contest	rationality–iteration	g6Riter
Game 7	ultimatum game	rationality–iteration	g7Riter
Game 8	maximize revenue	rationality–optimization	g8Ropti
Game 9	forward induction	all	g91, g92, g93, g94
Game 10	centipede game	all	g101, g102, g103

### 2.4. Participants

One hundred and eighty-seven subjects participated in this research (83 females). All of them were students of the University of Modena and Reggio Emilia, with an average age of 22.63 years (standard deviation: 3.87, min 17, max 48). Most of them were unmarried (174), have brothers or sisters (147) and have no sons or daughters (183). About one third of the participants' parents completed collage (58 fathers and 52 mothers). Twenty-six of them earned a perfect high-school score, while 30 out of the 75 (40%) who earned a Bachelor's Degree obtained a 110 or 110 cum laude score. The monthly income of the household was reported as being less than 1,000 euro for 9 of them, among 1,000 and 2,000 euro for 58 of them, among 2,000 and 2,500 euro for 69 of them, and more than 3,500 euro for 50 of them. Only 10 of them live alone, and only 32 of them live in a house owned by their family. Forty-five of them have had a stable job during the 6 months before participating to this research. Most of the participants were right-handed (161). All participants received recognition for completing the tests and could access the results of the tests taken, if requested. While traditionally in experimental economics subjects are incentivized with monetary rewards, we followed the psychological tradition of avoiding external motivators. Participants were recruited via academic e-mail communication and were briefed about the scopes and modalities of the tests and participation. All participants signed informed consent. Participants were completely voluntary and could drop out at any time without any negative consequence. Informed consent was obtained from all participants. All data were stored only using an anonymous ID for each participant. The study reported in this manuscript has been conducted in accordance with the practices for non-clinical research of the ethic committee of the University of Modena and Reggio Emilia, and includes non-clinical tests using non-invasive, non-harmful measures. No treatment, false feedback or deceiving method was used. Accordingly, we followed the ESOMAR guidelines^3^ stressing anonymity, privacy, and voluntariness of participants. According to the local and national and guidelines this kind of research does not require full review and approval by the ethic committee.

## 3. Results

### 3.1. Game Scores and Their Correlations

All game scores were computed endogenously, meaning that the score obtained by each participant in each game was calculated using the other participants' play for the same game. This was made clear to all participants at the beginning of the test, and emphasized in the written instructions given as reference. Game scores were aggregated in 12 score variables for the first eight games, and in 7 variables for Game 9 and Game 10, following the lines described in section 2.3. Table 2 reports summary statistics for game score variables.

**Table 2:** Summary statistics of the game score variables for Game 1 to 10.

**Game score variable**	**Obs**	**Mean**	**Std. Dev**.	**Min**	**Max**
g1Mpref	187	0.36	0.07	0.12	0.51
g2Mpref	187	0.38	0.07	0	0.53
g5Mpref	187	0.72	0.33	0	1
g1Mskil	187	0.27	0.08	0.03	0.42
g2Mskil	187	0.28	0.06	0	0.41
g5Mskil	187	0.86	0.33	0	1
g3Ropti	187	0.96	0.18	0	1
g4Ropti	187	0.94	0.17	0	1
g8Ropti	187	0.60	0.26	0	1
g4Riter	187	0.69	0.34	0	1
g6Riter	187	0.52	0.19	0	0.99
g7Riter	187	0.54	0.25	0	1
g91	187	0.20	0.40	0	1
g92	187	0.52	0.50	0	1
g93	187	0.61	0.49	0	1
g94	187	0.31	0.46	0	1
g101	187	0.20	0.40	0	1
g102	187	0.37	0.48	0	1
g103	187	0.25	0.43	0	1

Table 3 reports the pairwise correlations among all these score variables which resulted statistically significant at least at the 5% level. The small number of significant correlations across the four groups of score variables for Game 1 to 8 suggests that such groups actually capture different correlates of strategic decision-making. Reasonable exceptions are *g2Mpref* and *g2Mskil*, which are presumably correlated because they record scores of similar tasks in Game 2, and *g1Mskil* with all variables for *Riter*, which may be due to the fact that good knowledge of others' skills correlates with basic cognitive skills which in turn are a prerequisite for iterative rationality. This is confirmed by correlation with Raven scores (0.24, *p* < 0.001). The significant correlation between *g3Ropti* and *g4Riter* is less straightforward. One possibility is that, given the low difficulty to be successful in Game 3, *g3Ropti* also captures complete failure to understand the sequential game structure, which in turn is a prerequisite for a high score in *g4Riter* (and not for *g4Ropti*).

**Table 3:** Pairwise correlations of all variables recording game scores.

	**g1Mpref**	**g2Mpref**	**g5Mpref**	**g1Mskil**	**g2Mskil**	**g5Mskil**	**g3Ropti**	**g4Ropti**	**g8Ropti**	**g4Riter**	**g6Riter**	**g7Riter**	**g91**	**g92**	**g93**	**g94**	**g101**	**g102**	**g103**
g1Mpref	1.00																		
g2Mpref	0.2347	1.00																	
	(0.0012)																		
g5Mpref			1.00																
g1Mskil				1.00															
g2Mskil		0.2605		0.5314	1.00														
		(0.0003)		(0.0000)															
g5Mskil						1.00													
g3Ropti							1.00												
g4Ropti								1.00											
g8Ropti							0.1663		1.00										
							(0.0233)												
g4Riter				0.2892			0.1627			1.00									
				(0.0001)			(0.0261)												
g6Riter				0.1581						0.3479	1.00								
				(0.0307)						(0.0000)									
g7Riter				0.1787						0.2043		1.00							
				(0.0144)						(0.0050)									
g91											0.1437	0.1663	1.00						
											(0.0498)	(0.0229)							
g92	0.1419		0.1525	0.2378	0.1604		0.1826			0.2954	0.2572	0.1510	0.3003	1.00					
	(0.0527)		(0.0372)	(0.0010)	(0.0283)		(0.0124)			(0.0000)	(0.0004)	(0.0391)	(0.0000)						
g93						0.1542						0.2084	0.2716		1.00				
						(0.0351)						(0.0042)	(0.0002)						
g94										0.1987	0.2221	0.5520	0.3220		0.2692	1.00			
										(0.0064)	(0.0022)	(0.0000)	(0.0000)		(0.0002)				
g101	0.1716		-0.1655			-0.1546				0.1464							1.00		
	(0.0188)		(0.0236)			(0.0346)				(0.0456)									
g102								0.1754	0.1688									1.00	
								(0.0163)	(0.0209)										
g103																			1.00

*Only correlations which are statistically significant at least at the 5% level are reported (p-values are in parentheses)*.

In the group of variables for *Mpref* we find substantial correlation between *g1Mpref* and *g2Mpref*. Moreover, *g1Mpref* is significantly correlated with score variables for Game 9 and 10. In the group of variables for *Mskil* we find strong correlation between *g1Mskil* and *g2Mskil*. Moreover, both *g1Mskil* and *g2Mskil* are both significantly correlated with a score variable for Game 9. In the group of variables for *Ropti* we find significant correlation between *g3Ropti* and *g8Ropti*. Further, *g3Ropti* is significantly correlated with a score variable for Game 9, while *g4Ropti* and *g8Ropti* are significantly correlated with a score variable for Game 10. In the group of variables for *Riter* we find significant correlation between *g4Riter* and both *g6Riter* and *g7Riter*. Also, all score variables in this group correlate significantly with score variables for Game 9 and *g4Riter* also correlates with a score variable for Game 10.

Score variables for Game 5, i.e., *g5Mpref* and *g5Mskil*, and *g4Ropti* do not correlate significantly with any other score variable for Game 1 to 8. Moreover, while *g4Ropti* correlates positively with a score variable for Game 10, both *g5Mpref* and *g5Mskil* do correlate positively with a score variable for Game 9 but they also correlate negatively with a score variable for Game 10. Remarkably, these are the only negative correlations which are statistically significant at the 5% level. If, on the one hand, this suggests that the recorded score variables have a common underlying determinants, on the other hand, this also suggests that score variables for Game 5 may not capture well any of the hypothesized components of mentalization and rationality.

Finally, the stark contrast between the rarity of significant correlations among score variables for Game 1 to 8 and the abundancy of significant correlations between score variables for Game 1 to 8 and score variables for Game 9 and Game 10 is consistent with the idea underlying the design of our test: playing successfully Game 9 and Game 10 involves several dimensions of strategic ability which are individually involved to play successfully Game 1 to 8. In this regard, we can also ask to what extent Games 1 to 8 have been successfully designed to capture distinct dimensions of strategic abilities. One way to check this is to look at Cronbach's α which is a measure of the reliability of a test with many items attempting to measure one single construct. If we designed the Games 1–8 well, then α is bound to be very low. Indeed, it turns out that α = 0.36 when we consider all score variables, which suggests that this would not be a reliable test to measure a single latent variable.

### 3.2. Extraction of Factors Form Scores in Game 1 to 8

For each group of variables described in section 2.3 we ran a principal component analysis. We only took the orthogonally rotated first component for each set of variables as the factor associated with each group of variables. All factors have been standardized. Table 4 reports the relevant information for the factors extracted. We call *Mpref* the principal component of *g1Mpref*, *g2Mpref*, and *g5Mpref*, we call *Mskil* the principal component of *g1Mskil, g2Mskil*, and *g5Mskil*, we call *Ropti* the principal component of *g3Ropti, g4Ropti*, and *g8Ropti*, and finally we call *Riter* the principal component of *g4Riter, g6Riter*, and *g7Riter*.

**Table 4:** The four factors extracted as first components from distinct set of variables.

**N. of individuals = 187**	**Mpref**	**Mskil**	**Ropti**	**Riter**
Eigenvalue	1.24445	1.53946	1.1771	1.50277
Variance explained	41.48%	51.32%	39.24%	50.09%
g1Mpref	**0.6743**			
g2Mpref	**0.7112**			
g5Mpref	–0.1989			
g1Mskil		**0.7057**		
g2Mskil		**0.6980**		
g5Mskil		0.1216		
g3Ropti			**0.7304**	
g4Ropti			**0.2569**	
g8Ropti			**0.6329**	
g4Riter				**0.6223**
g6Riter				**0.6142**
g7Riter				**0.4853**

*Component loadings of 0.25 or higher were considered significant and are in bold*.

Each first component captures a large part of the overall variance of the associated game score variables. Each component is associated with a sizeable eigenvalue. However, while for the rationality factors *Ropti* and *Riter* the factor loadings are all positive and greater than 0.25, both *Mpref* and *Mskil* fail to capture the variance of the two variables recording player's success in Game 5 (*gM5pref* and *gM5skil*, respectively). This means that *Mpref* and *Mskill* do not convey much information underlying the decisions made in Game 5. In the light of the pairwise correlations discussed in section 3.1 this is not necessarily undesirable, although it is a further indication that score variables for Game 5 may not capture well any of the hypothesized components of mentalization and rationality.

One perhaps surprising result is the very low correlation between *Ropti* and *Riter*. Indeed, one may expect that the ability to solve simple optimization problems in a given environment is related to the more sophisticated iterative reasoning where the actual environment has also to be determined. However, this does not necessarily lead to large correlation coefficients if most people are actually good in doing simple optimizations while few people are good in doing iterative reasoning, as it is the case in our sample (see Table 1).

Table 5 reports the pairwise correlations between the four factors extracted. Since each factor comes from a distinct set of variables there is no necessity for them to be orthogonal. Nevertheless, correlations are small and hardly statistically significant at 5% level, indicating that the four factors capture distinct dimensions underlying the recorded measures of strategic success. These correlations, together with the factor loadings in Table 1, seem consistent with the theoretical framework presented in section 2.1.

**Table 5:** Pairwise correlations of the four extracted components.

	**Mpref**	**Mskil**	**Ropti**	**Riter**
Mpref	1.00			
Mskil	0.15^*^	1.00		
	(0.04)			
Ropti	–0.10	0.05	1.00	
	(0.18)	(0.49)		
Riter	0.06	0.22^**^	0.05	1.00
	(0.39)	(0.01)	(0.51)	

*The p-values are reported in parentheses*.

### 3.3. Theory-Driven Analysis vs. Data-Driven Analysis

In our analysis we have identified the four factors by grouping score variables in four different sets from which we have extracted the first component. This is a theory-driven approach. To check its validity we also carried out two additional principal component analyses which are more data-driven. In the first analysis score variables are grouped in two sets, one group for rationality and one for mentalization, and extract the first two components for each. This analysis only presumes a relevant role for the two main dimensions. The set of variables associated with rationality is the union of the sets of variables used to extract *Ropti* and *Riter*, while the set of variables associated with mentalization is the union of the sets of variables used to extract *Mpref* and *Mskil*. In the second principal component analysis all score variables are grouped in a single set from which four components are extracted. This analysis is fully data-driven.

Results are reported in Table 6 and are remarkable. The two factors extracted from the group of score variables for rationality are statistically indistinguishable from *Ropti* and *Riter* (significant correlation of 0.99 and 0.96, respectively). In particular, factor 1 resembles *Riter*, accounts for 26% of the variance and has an associated eigenvalue of 1.55, while factor 2 resembles *Ropti*, accounts for 20% of the variance and has an associated eigenvalue of 1.19. The two factors extracted from the group of score variables for mentalization are statistically indistinguishable from *Mpref* and *Mskil* (significant correlation of 0.95 and 0.97, respectively). In particular, factor 1 resembles *Mskil*, accounts for 26% of the variance and has an associated eigenvalue of 1.58, while factor 2 resembles *Mpref*, accounts for 21% of the variance and has an associated eigenvalue of 1.19. In short, the two components hypothesized for mentalization and rationality also emerge, and basically coincide, with the factors extracted with the theory-driven analysis presented in section 3.

**Table 6:** Comparison with more data-driven PCA.

**First factor from**	**Factor with highest correlation extracted from**					
**4 groups of var**	**2 groups of var**			**1 group of var**		
**Mentalization**	**Mentalization**	**Eigenvalue**	**Variance**		**Eigenvalue**	**Variance**
Preference	Factor 2	1.30285	21%	Factor 3	1.35593	12%
Mpref	0.9492			0.7550		
*p*-value	<0.001			<0.001		
Skills	Factor 1	1.58062	26%	Factor 1	1.64267	15%
Mskil	0.9863			0.9145		
*p*-value	<0.001			<0.001		
**Rationality**	**Rationality**	**Eigenvalue**	**Variance**		**Eigenvalue**	**Variance**
Optimization	Factor 2	1.19356	20%	Factor 4	1.23235	11%
Ropti	0.9865			0.4510		
*p*-value	<0.001			<0.001		
Iteration	Factor 1	1.55072	26%	Factor 2	1.58839	14%
Riter	0.9610			0.9561		
*p*-value	<0.001			<0.001		

*The left column indicates the factor extracted from the set of variables described in section 2.3. The central column reports correlation coefficients with pairs of factors extracted from two sets of variables (one for Mentalization, containing all variables used to extract Mpref and Mskil, one for Rationality containing all variables used to extract Ropti and Riter). Only the highest correlation coefficient is reported, with the associated factor number (denoting the order of extraction). The right column reports correlation coefficients with four factors extracted from a single set containing all variables (again, the factor number denotes the order of extraction)*.

The four factors extracted from the group made of all score variables are extremely close to *Mpref*, *Mskil, Ropti, Riter* (significant correlation of 0.76, 0.91, 0.45, and 0.97, respectively). In particular, factor 1 resembles *Mskil*, accounts for 15% of the overall variance and has an associated eigenvalue of 1.64, factor 2 resembles *Riter*, accounts for 14% of the overall variance and has an associated eigenvalue of 1.59, factor 3 resembles *Mpref*, accounts for 12% of the variance and has an associated eigenvalue of 1.36, while factor 4 resembles *Ropti*, accounts for 11% of the variance and has an associated eigenvalue of 1.23. In short, of the four components that we extracted from a fully data-driven analysis, two basically coincide with *Mskil* and *Riter*, one component is extremely close to *Mpref* and one component substantially correlates with *Ropti*. Overall, we find that our theory-driven approach is well supported by data-driven principal component analysis.

### 3.4. Synthetic Measures of the Strategic Quotient

The findings presented so far suggest that, by adequately measuring an individual's abilities related to the cognitive dimensions of mentalization and rationality, we can try to assess the overall strategic ability of such individual. This in turn suggests that we can attempt to construct a synthetic measure of strategic ability, the Strategic Quotient (SQ), building on reliable measures of mentalization and rationality. As a first rough approximation we can sum up the scores obtained for Game 1 to 8 in a single variable, denoted with *SQ8*. This variable is likely to contain valuable information on both mentalization and rationality since the scores for Game 1 to 8 have been effectively used to extract the factors related to mentalization, *Mpref* and *Mskil*, and those related with rationality, *Ropti* and *Riter*. As a less rough measure of the SQ we can sum up these four factors in a single variable, *SQfactor*. This variable should be strongly correlated with *SQ8* while taking away a substantial part of confounding factors present in game score variables.

**Figure 1:**
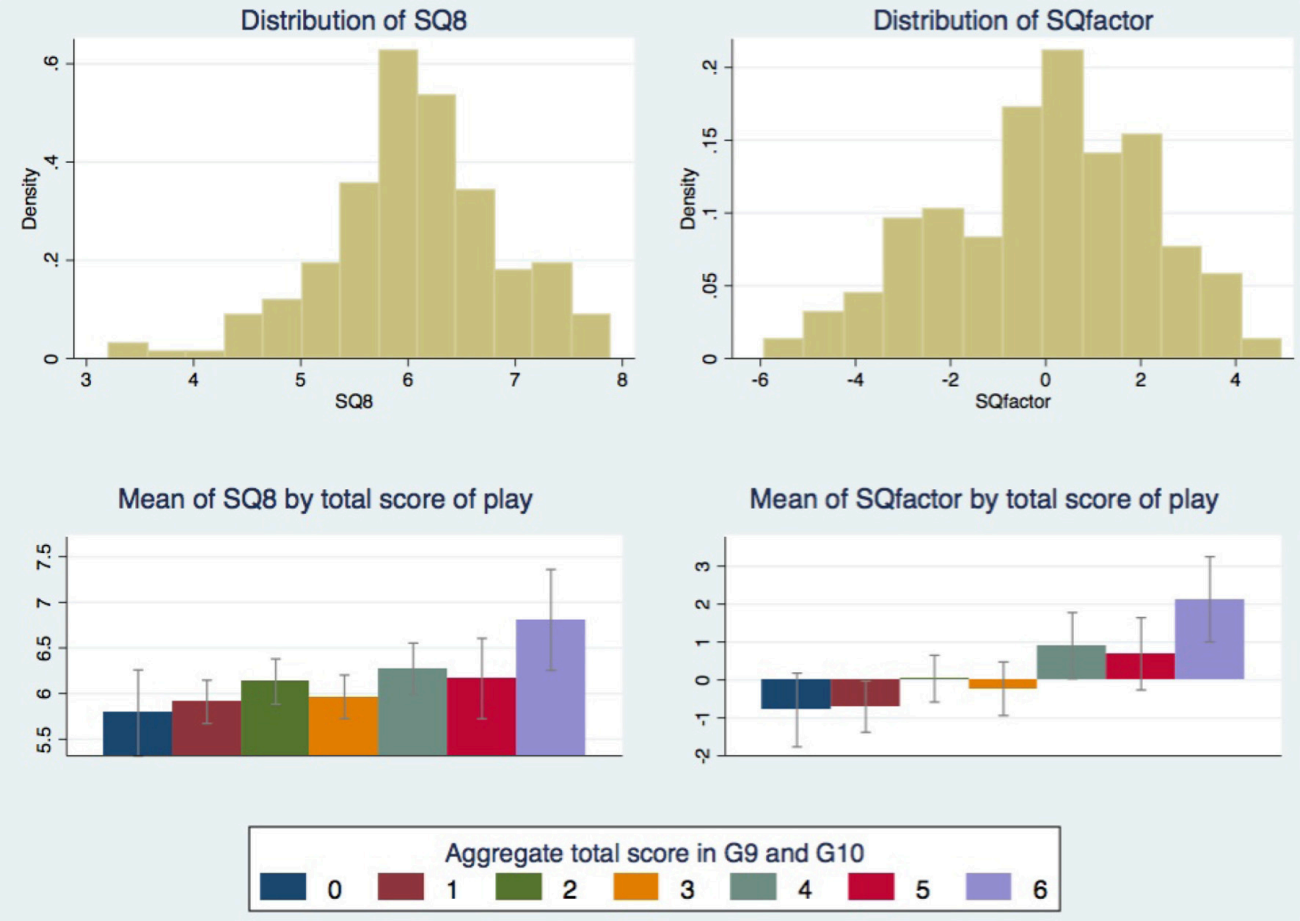
**(Top)** Distribution of the sum of scores in Game 1 to 8 (variable *SQ8*) and the sum of *Mpref*, *Mskil, Ropti*, and *Riter* (variable *SQfactor*). **(Bottom)** Means of SQ measures (of variable *SQ8* on the left and of variable *SQfactor* on the right) conditional on aggregate total scores in Game 9 and Game 10. Confidence intervals are at 95% level.

A first look at the distribution of *SQ8* and *SQfactor* indicate that there are no ceiling or floor effects and that both are single peaked for a mid value (around 6 for *SQ8* and around 0.02 for *SQfactor*). The distributions of the two variables are depicted in the upper charts of Figure 1. As expected, the correlation between *SQ8* and *SQfactor* is very high, 0.7754, and statistically significant (*p* < 0.001). Hartigan's dip test statistic provides support for unimodality for both SQ8 and SQfactor variables (respectively: 0.0179, *p* > 0.97, and 0.0169, *p* > 0.99).

The next step is to check to what extent *SQ8* and *SQfactor* can account for the overall strategic success in Game 9 and Game 10. To do this we aggregate the scores in Game 9 and Game 10 by summing up *g91, g92, g93, g94, g101, g102*, and *g103*, obtaining a single variable ranging from 0 (always unsuccessful) to 7 (always successful). The correlation between SQ measures and the aggregate score variable is strong and statistically significant. More precisely, the correlation with *SQ8* is 0.20 with *p*-value 0.0045 while the correlation with *SQfactor* is 0.30 with *p* < 0.001. So, both SQ measures positively correlate with overall strategic success of a player, with *SQfactor* being sensibly more correlated.

To better understand the relation between SQ measures and the degree of strategic success in Game 9 and Game 10, we calculate means of *SQ8* and *SQfactor* conditional on the aggregate scores in these games. The lower charts of Figure 1 shows the conditional means. Although all means of *SQ8* are between 5.8 and 6.8, the mean of *SQ8* conditional on a score of 6 (the maximum observed) is more than 0.5 higher than the mean of *SQ8* conditional on a score of 5, which in turn is about 0.5 higher than the mean of *SQ8* conditional on a score of 0 (the minimum observed). A similar pattern is obtained for the conditional means of *SQfactor*. Such evidence suggests that these rough measures of SQ mostly capture the top strategic performances in both Game 9 and Game 10.

We further investigate the predictive power of our SQ measures by running OLS regressions where the dependent variable is the sum of scores in Game 9 and Game 10 and, alternatively, *SQ8* or *SQfactor* is the independent variables, possibly with a number of controls. Table 7 reports the estimates. In regressions (1) and (2) the only regressors are, respectively, *SQ8* or *SQfactor*, while in regressions (3) and (4) we also include the following individual-level control variables: gender (1 = Male, 0 = Female), net monthly income (1 = less than 1,000 euro, 2 = between 1,000 and 2,000 euro, 3 = between 2,000 and 3,500 euro, 4 = more than 3,500 euro), age, college completion (1 = if obtained a college degree, 0 = if not), mother and father college completion (1 = if obtained a college degree, 0 = if not). Estimates indicate that one standard deviation increase in *SQ8* (0.95) goes with an extra 0.38 of overall score in Game 9 and 10 (equal to 0.25 of its standard deviation) and, similarly, one standard deviation increase in *SQfactor* (2.21) goes with an extra 0.44 of overall score (equal to 0.28 of its standard deviation).

**Table 7:** OLS regressions.

**Scores in Game 9 and 10 predicted by SQ measures**				
	**(1)**	**(2)**	**(3)**	**(4)**
SQ8	0.3901^**^		0.4142^**^	
	(0.1213)		(0.1245)	
SQfactor		0.2159^*^		0.1909^**^
		(0.0487)		(0.0508)
Male			0.7481^**^	0.6143^**^
			(0.2196)	(0.2211)
Monthly income			0.0296	0.0535
			(0.1378)	(0.1355)
Age			–0.0450	–0.0364
			(0.0281)	(0.0285)
Completed college			–0.2441	–0.2294
			(0.2356)	(0.2369)
Father completed college			–0.0844	–0.1050
			(0.2885)	(0.2888)
Mother completed college			0.2452	0.2128
			(0.3309)	(0.3222)
Observations	187	187	186	186
Individual controls	No	No	Yes	Yes

Dependent variable is the total score in Game 9 and Game 10. Statistical significance is denoted by

** if p-value < 0.01 and

* *p-value < 0.05. Robust standard errors are in parentheses*.

### 3.5. Relation With Other Economic, Educational, and Psychological Variables

A further inquiry of potential interest that our rich dataset allows is the comparison between our SQ measures and economic, educational, and psychological variables. More precisely, we considered monthly income, college completion, attending lyceum high school, our recorded Raven score, our 3-item IQ measure in the pre-test, the 6-item CRT score, measures of rational attitude and experiential attitude, as captured by the REI40 (Pacini and Epstein, 1999), and measures of personality traits, as measured with the 46 items BIG5 inventory (John et al., 1991). Since this analysis is explorative p-values are adjusted for multiple testing in the most conservative way (Bonferroni). As a result we find that the only statistically significant correlate of *SQ8* is the CRT score (0.27, *p* < 0.0176), while the only statistically significant correlates of *SQfactor* are CRT score (0.33, *p* < 0.0003) and our our 3-item IQ measure in the pre-test (0.29, *p* < 0.0053). This suggests that while our SQ measures and other measures of cognitive abilities may reasonably overlap to some extent, they do capture different things. Moreover, the significant correlation with CRT is consistent with the idea that the ability to overrule intuitive decisions with more reflective ones plays an important role in strategic thinking.

Further, the fact that we found no statistically significant correlation between either measure of SQ and the BIG five personality traits suggests that SQ measures, which are aimed at capturing characteristics related to the way individuals reason and make decision, may not be much affected by characteristics of personality. Importantly, this remains true even if we look at the correlations of personality traits with the single factors *Mpref*, *Mskil, Ropti*, and *Riter*.

We also investigate if the predictive power of our SQ measures for the success in Game 9 and 10 is reduced when we also include the variables considered in this subsection as controls in the OLS regressions presented in Table 7. Estimated coefficients are reported in Table 8. Remarkably, both *SQ8* and *SQfactor* maintain a significant predictive power, while all the newly included variables have non-significant coefficients. In particular, one standard deviation increase in *SQ8* goes with an extra 0.27 of overall score in Game 9 and 10 while one standard deviation increase in *SQfactor* (2.21) goes with an extra 0.28 of overall score.

**Table 8:** OLS regressions.

**Scores in Game 9 and 10 predicted by SQ measures**		
	**(1)**	**(2)**
SQ8	0.2891^*^	
	(0.1336)	
SQfactor		0.1334^*^
		(0.0571)
completed lyceaum	0.2647	0.2489
	(0.4331)	(0.4441)
rational_attitude	0.4124	0.3834
	(0.2633)	(0.2646)
experiential_attitude	–0.2704	–0.2251
	(0.2486)	(0.2511)
CRT6	0.1057	0.1079
	(0.0778)	(0.0786)
Raven APM score	–0.0459	–0.0434
	(0.0265)	(0.0287)
3-item IQ	0.0169	–0.0172
	(0.1894)	(0.1936)
Extroversion	–0.1090	-0.1602
	(0.1532)	(0.1512)
Agreeableness	0.1728	0.2011
	(0.2015)	(0.1994)
Conscientiousness	–0.2682	–0.2197
	(0.1632)	(0.1635)
Neuroticism	–0.0110	–0.0132
	(0.1795)	(0.1767)
Openess	0.0708	0.0750
	(0.2105)	(0.2080)
Observations	186	186
Individual controls	Yes	Yes

Dependent variable is the total score in Game 9 and Game 10. Controls of regressions (3) and (4) in Table 7 are also included but not reported. Statistical significance is denoted by ^**^ if p-value < 0.01 and

* *p-value < 0.05. Robust standard errors are in parentheses*.

### 3.6. Relation With Models of Strategic Sophistication

The literature on behavioral game theory has developed formal models of strategic sophistication which have the common feature of measuring the degree of sophistication in terms of what is called the *depth of reasoning*. In the level-*k* model of reasoning (Stahl and Wilson, 1994, 1995; Nagel, 1995) a level-*k* type of player best responds by assuming that all other agents are level-(*k* − 1) types. The parameter *k* is a measure of the degree of strategic sophistication in that it counts the number of iterations of best-reply reasoning to an initial belief (typically that other players are all level-0 and play randomly). In the cognitive hierarchy model (Camerer et al., 2004; Chong et al., 2006) level-*k* reasoning is modeled with greater flexibility by letting a level-*k* type best respond to a distribution of types from level-0 to level-(*k* − 1). The endogenous depth of reasoning model (Alaoui and Penta, 2016, 2018) assumes that the actual depth of reasoning of a player, e.g., the number of iterations of best-reply reasoning to an initial belief, is not exogenously given but the result of a decision-making process that depends on cognitive abilities and payoffs.

Our dataset has not been designed for the purpose of allowing a comparative evaluation of the predictive power of these models [see Arjona et al., (unpublished); Alós-Ferrer et al., 2018 for dedicated attempts] but it is rich enough to provide a simple and clean proxy of the actual depth of reasoning of participants: the number between 1 and 90 played against the automaton in Game 6 (i.e., the third task in Game 6, for which the only correct answer is 1 and whose score contributes to 1/3 of the score variable *g6Riter*). More precisely, a smaller number is a proxy for a greater depth of reasoning. Note that, since in this strategic interaction the opponent is an automaton whose strategy is known in advance, if the participant correctly understands the instruction then his actual decision is independent of initial subjective beliefs and only depends on the ability to iterate best responses.

We constructed a variable measuring the depth of reasoning, *DoR*, by taking the natural logarithm of the difference between 90 and the number played against the automaton in Game 6. Interestingly, and perhaps not too surprisingly, the variable *DoR* is strongly and significantly (with Bonferroni correction for testing over all factors extracted) correlated with *Riter* (0.40, *p* < 0.0001) and *Mskil* (0.22, *p* < 0.03) but not significantly correlated with *Mpref* and *Ropti*. As a consequence, *DoR* strongly and significantly correlates with both *SQ8* and *SQfactor* (respectively: 0.23, *p* < 0.004, and 0.32, *p* < 0.0001) and is a good predictor of overall success in Games 9 and 10, with a correlation of 0.29 (*p* < 0.001). Differently from *SQ8* and *SQfactor*, the distribution of *DoR* has a single peak at its maximum value (the deepest reasoning level). Moreover, while *SQ8* and *SQfactor* seem to discriminate well especially for extreme score values (as can be seen in the bottom charts of Figure 1), *DoR* seems to discriminate well especially for mid score values (the mean of *DoR* conditional on the overall score in Game 9 and 10 varies between 3.9 and 4.4 with a jump of about 0.3 passing from a score of 2 to a score of 3).

## 4. Discussion

### 4.1. Conclusions

Success in competitive settings is, ceteris paribus, mainly driven by strategic ability. A reliable measure of strategic ability, which is currently missing, would be useful to predict success in competitions. In this research we have moved the first step toward the construction of a such a measure, the “Strategic Quotient” (SQ). To this aim, we designed and administered a test where each item is a game. The main novelty of our test lies in the fact that the score obtained in each item depends on the distribution of decisions made by other decision-makers taking the test. From the collected data we have extracted information on the abilities related to two dimensions that we argue are fundamental to strategic success: mentalization and rationality. For each of these dimensions we have identified two main factors worth measuring: understanding of others' preferences and understanding of others' cognitive skills (mentalization); and capacity to optimize as well as capacity to iterate reasoning (rationality). The evidence provided by the collected data suggests that these four factors well account for strategic success in most of the simpler games that we have designed to involve mostly either a factor of mentalization or one of rationality; moreover, the same data suggest that these factors relate to strategic success in two more sophisticated games designed to involve crucially both mentalization and rationality. Finally, collected data show that aggregate synthetic measures built on our test, summing up either game scores or extracted factors, can provide information on the likelihood of strategic success in the two more sophisticated games. Overall, this supports the possibility of building a synthetic measure of strategic abilities using only simple games designed to capture information about a single factor underlying mentalization and rationality.

### 4.2. Limitations and Future Research

The research reported in this paper should be considered with the following limitations in mind. Our sample is entirely of university students enrolled at the University of Modena and Reggio Emilia, and therefore it is in no way representative of the overall population. In particular, given the endogenous nature of game scores, this limits the interpretation of strategic success as success when playing against other students. So, our claims regarding the measurement of strategic abilities should be interpreted accordingly. However, in our opinion this limitation is necessary in the current first steps of this research, because we need to give to players an idea of who are the other players in order to assess their ability to mentalize. More in general, any approach which, like ours, tries to measure strategic success by inferring it from actual strategic behavior necessarily leads to a context-dependent measure of strategic ability because scores depend on the group of players actually considered. In this regard an important research step to be done is to identify a number of broad and relevant contexts (i.e., type of participants such as school students, college students, self-employed, workers, elder, etc.) to get meaningful distributions across populations. This would allow to have standard reliable distributions for the purpose of measuring strategic abilities in relevant contexts.

Another limitation of this research is the small number and low variety of games involving more than one dimension of strategic ability. In our study only Game 9 and Game 10 are designed in such a way that strategic success reasonably involves more than one component of either mentalization or rationality. In particular, success in Game 9 (that may require the player to use and assess the opponent's use of forward induction) reasonably involves mostly rationality, especially in the form of iteration, and mentalization, especially in the form of knowledge of others' skill; instead, Game 10 (that may require the player to use backward induction as well as to assess others' preference for a fair divisions of prizes or for social efficiency) reasonably involves mostly rationality, especially in the form of iteration, and mentalization, especially in the form of knowledge of others' preferences. Besides these two games, many others are both interesting and relevant for real strategic interactions such as auctions, social dilemmas, war of attrition, bargaining, voting games, and coalitional games. Many of these should be considered in order to assess the validity of a general measure of strategic ability.

Further, the collected data point to a few potentially important modification of the proposed test. A first modification is the elimination of Game 5, perhaps replaced by a game that captures mentalization with less confounding factors. Indeed, Game 5 contains two pure coordination games: one about coordinating on a color, where we supposed that knowledge of others' preferences would have been crucial, and one about coordinating on number with the provision of a focal point on the number 12, where we supposed that knowledge of others' cognitive skills would have been crucial. Collected data seem to suggest that something else is crucial in such coordination games. Although this might be interesting in itself, it also suggests to eliminate or replace Game 5. Another game that should be modified is Game 3, because of an evident ceiling effect. Indeed, 92% of players got the maximum payoff in this game, suggesting that it mostly discriminates mistakes, misunderstandings and only very low optimization (rationality) ability. Also, a modification of Game 8 might be considered. Although success in this game seems to be well captured by *Ropti*, it also structurally involves knowledge of others' preferences (in particular, willingness to pay for taking the test). In the current analysis we could control for this since we also elicit such knowledge with Game 1 and Game 2, using it to extract *Mpref*. However, since this may not be feasible in some situations (e.g., in a test designed to elicit different preferences) it may be desirable to modify Game 8 in such a way that the knowledge of others' preference is less important for strategic success. Finally, the last part of Game 10 seems to have not been correctly understood by many players, suggesting that it should be replaced or, at least, better explained. Despite the relative simplicity of the iterative reasoning involved in this game (since the opponent is an automaton which never makes mistakes), only about 25% of the players were successful and many of those who where not successful made hardly rationalizable decisions. This could also explain why the score in this part of Game 10 was poorly predicted by our measures of mentalization and rationality.

Finally, we are aware that the administered test is quite long and effort-demanding for participants. This might have triggered someone to switch by a deliberative mode of reasoning to a more intuitive/heuristic one during the test, due to increased cognitive load or ego depletion (Evans, 2008; Glöckner and Witteman, 2010; Kahneman and Egan, 2011; Evans and Stanovich, 2013). This is a source of confounding effects that we might want to control for (for instance, by collecting response times; see, e.g., Alós-Ferrer et al., 2018)^4^.

Drawing conclusions, and in line with other contributions looking at the relationship between rationality and success (see, e.g., Javarone, 2015), our results indicate that rationality is not enough to ensure success in competitive settings if it is not coupled with mentalization. This observation may open a research line investigating (by means of analytical or computational techniques) the actual distribution of skills between rationality and mentalization as the result of evolutionary selection when the overall amount of skills is scarce. In pursuing this research, an important role is likely to be played by the degree of competitiveness, which might be seen as the number of competitors for a given stake (see, e.g., Javarone and Atzeni, 2015).

In future research we should also head for the development of a more parsimonious version of the test that is both short in terms of the time needed to administer it and easy in terms of numeracy and literacy competences needed. This might also help to keep experimental subjects in a deliberative mode of reasoning throughout the test. In this regard, we think we should aim for a test like the Cognitive Reflection Test (Frederick, 2005), which can be easily integrated in many experiments and surveys, allowing the construction of a vast database that can be used to assess strategic abilities in many distinct contexts. For instance, if further research confirms our finding that iterative reasoning is strongly correlated with actual play in a beauty contest game against an automaton that is known to always best reply, then iterative reasoning could be measured by a single item which score could be calculated without any need to consider other participants' play.

## Author Contributions

EB and LB contributed to the conception of the study and the early design of the test. AM tested and amended the first versions of games and instructions in order to make them easier to understand. All authors participated to data collection and organization. EB performed the statistical analysis. All authors contributed to the theoretical framework and to the interpretation of results. All authors contributed to manuscript writing and revision, read and approved the submitted version.

### Conflict of Interest Statement

The authors declare that the research was conducted in the absence of any commercial or financial relationships that could be construed as a potential conflict of interest.

## Footnotes

^1^A shortcut often used by economists to model scarcity of cognitive resources is to assume that individuals may use them but only at a cost (see Dewatripont and Tirole, 2005; Bilancini and Boncinelli, 2018a,b,c for recent applications).

^2^The inability to follow a good evaluation with an equally good decision has been called dysrationalia (Stanovich, 1993).

^3^https://www.esomar.org/publications-store/codes-guidelines.php

^4^Behavior has been shown experimentally to depend on the underlying mode of reasoning: see Rand et al. (2012), for prisoner dilemmas and public good games, Rand et al. (2016), for dictator games, Capraro (2017), for deception games, Capraro and Cococcioni (2015), for allocation decisions, Bilancini et al. (2017), for pure coordination games, Belloc et al., (unpublished), for stag-hunt games.

## Supplementary Material

Supplementary Presentation S1 Appendix.

Supplementary Data Sheet S1 SQ Test — Translated English version.

Supplementary Data Sheet S2 SQ Test — Original Italian version.
