# Supplementary material for: Assessing Actual Strategic Behavior to Construct a Measure of Strategic Ability
Source: Front Psychol. 2019 Jan 18;9:2750. doi: 10.3389/fpsyg.2018.02750 (PMC6345706; doi:10.3389/fpsyg.2018.02750)
Supplement: Supplementary Presentation S1 — Appendix. [file Presentation_1.pdf]

Appendix

A Numeric evaluations of answers

In the following we provide the details about how we compute aggregated numerical scores for an individual starting from his (and others') answers to the test and the pre-test.

We consider an individual participating to the test, and we denote with x a generic answer, and with x_{ki} the i-th answer of such individual in Game k. We use x_{Dki} to indicate the answer of the individual to Question Dki in the pre-test. With some abuse of notation, we also use x_{ki}^M and x_{51}^m to denote, respectively, the option that has been chosen by, respectively, the largest and the smallest fraction of participants as i-th answer in Game k. Finally, we use $|\cdot|$ to indicate the absolute value, I(condition) the indicator function (taking the value 1 if condition is true, 0 otherwise), and p(condition) to indicate the overall fraction of participants choosing an answer which satisfied condition.

For Game 9 and Game 10, we have assigned scores of either 0 or 1 to Game 9 (four scores: $g91$, $g92$, $g93$, $g94$) and Game 10 (three scores: $g101$, $g102$, $g103$), since such scores are then used as dependent variables in logistic regressions (see Appendix B). The specific assignments rules in the formulas reflect best reply behavior as it results from actual data.

Game 1. We denote with $\bar{x}_{1i}$ the answer in the pre-test corresponding to x_{1i}, averaged over all participants. Then:

$$g1Mpref = \frac{4}{7}\left(\left(1-\frac{|x_{11}-\bar{x}_{11}|}{100}\right)^2+\left(1-\frac{|x_{12}-\bar{x}_{12}|}{100}\right)^2+\left(1-\frac{|x_{13}-\bar{x}_{13}|}{100}\right)^2+\left(1-\frac{|x_{17}-\bar{x}_{17}|}{100}\right)^2\right).$$

$$g1Mskil = \frac{3}{7}\left(\left(1-\frac{|x_{14}-\bar{x}_{14}|}{100}\right)^2+\left(1-\frac{|x_{15}-\bar{x}_{15}|}{100}\right)^2+\left(1-\frac{|x_{16}-\bar{x}_{16}|}{100}\right)^2\right).$$

Game 2. We denote with $\bar{x}_{2i}$ the answer in the pre-test corresponding to x_{2i}, averaged over all participants. Then:

$$g2Mpref = \frac{4}{7}\left(\left(1-\frac{|x_{21}-\bar{x}_{21}|}{100}\right)^2+\left(1-\frac{|x_{22}-\bar{x}_{22}|}{100}\right)^2+\left(1-\frac{|x_{23}-\bar{x}_{23}|}{100}\right)^2+\left(1-\frac{|x_{27}-\bar{x}_{27}|}{100}\right)^2\right).$$

$$g2Mskil = \frac{3}{7}\left(\left(1-\frac{|x_{24}-\bar{x}_{24}|}{100}\right)^2+\left(1-\frac{|x_{25}-\bar{x}_{25}|}{100}\right)^2+\left(1-\frac{|x_{26}-\bar{x}_{26}|}{100}\right)^2\right).$$

Game 3.

$$g3_Ropti = \frac{1}{3}(I(x_{31}=E)+I(x_{32}=E)+I(x_{33}=E)).$$

Game 4.

$$g4Ropti = \frac{1}{3}\left(I(x_{42}=\text{corpo})+I(x_{44}=\text{testa})+I(x_{45}=\text{testa})\right).$$

$$g4Riter = \frac{1}{2}\left(\frac{P(x_{41})-P(x_{41}^m)}{P(x_{41}^M)-P(x_{41}^m)}+I(x_{46}=\text{fioretto})\right).$$

Game 5.

$$g5Mpref = \frac{p(x_{51}) - p(x_{51}^m)}{p(x_{51}^M) - p(x_{51}^m)}.$$

$$g5Mskil = \frac{p(x_{52}) - p(x_{52}^m)}{p(x_{52}^M) - p(x_{52}^m)}.$$

Game 6. We denote with y and $\tilde{y}$ the integer that is closest to 2/3 of the average number chosen by, respectively, all participants to the test and those who answered correctly to questions D1, D2 and D3 in the pre-test.

$$g6Riter = \frac{1}{3}\left(\frac{|x_{61} - y|}{90}^2 + \frac{|x_{62} - \tilde{y}|}{90}^2 + I(x_{63} = 1)\right).$$

Game 7.

$$g7Riter = \frac{1}{2}\left(I(x_{71} \leq 1) + \frac{(100 - x_{72})p(x_{D32} \leq x72)}{\max_x (100 - x)p(x_{D32} \leq x)}\right).$$

Game 8.

$$g8Ropti = \frac{x_{82}p(x_{D5} \geq x82)}{\max_x xp(x_{D5} \geq x)}.$$

Game 9.

$$g91 = I(x_{91} = \text{S} \ \& \ x_{92} = \text{Caricare}).$$

$$g92 = I(x_{93} = \text{Ritirarsi}).$$

$$g93 = I(x_{94} = \text{Caricare}).$$

$$g94 = I(x_{95} = \text{S} \ \& \ x_{96} = \text{Caricare}).$$

Game 10.

$$g101 = I(x_{101} = \text{Termina allo stadio 3}).$$

$$g102 = I(x_{102} = \text{Termina allo stadio 4}).$$

$$g103 = I(x_{103} = \text{Termina allo stadio 1}).$$

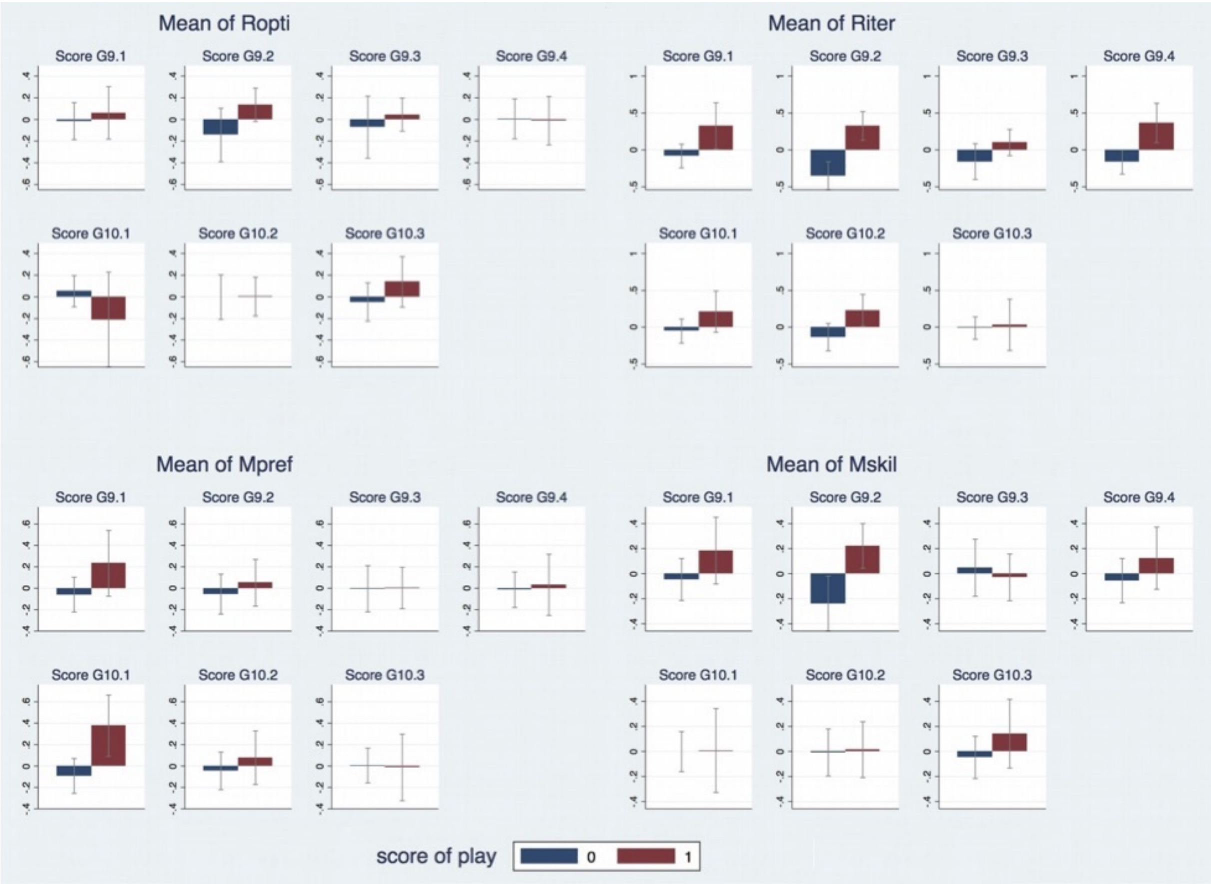

Figure 1: Mean of *Mpref* conditional on game scores (1 is success) in Game 9 and Game 10. Confidence intervals are at 95%.

B Relation of single factors with scores in Game 9 and 10

To study the relation between each factor and the score variables for Game 9 and Game 10 we proceed in two steps. First, we look at the mean of each factor conditional on strategic success as coded by each score variable for Game 9 and Game 10. Second, we run logit regression and calculate marginal effects to quantify how factors jointly predict the likelihood of success in Game 9 and Game 10.

B.1 Factor means by score variables

We calculate the means of *Mpref*, *Mskil*, *Ropti* and *Riter* conditional the score of g91, g92, g92, g94, g101, g102, and g103. The difference between the mean conditional on success and the mean conditional on failure is statistically significant at 5% level in a number of cases (as can be understood from Figure 1). However, if we take a more conservative perspective correcting statistical significance for multiple testing (Bonferroni), then most differences become non-significant. A regression analysis seem to be required to explore the prediction power of single factors for games scores in Game 9 and 10.

B.2 Marginal effects in logistic regressions

We run a number of logit regressions with dependent variable, in turn, a score variable of Game 9 and Game 10, namely g91, g92, g93, g94, g101, g102, and g103. For each of these score variables we run two distinct logit regressions. In the first regression the independent variables are the four factors extracted from score variables for Game 1 to 8, namely *Mpref*, *Mskil*, *Ropti*, and *Riter*. In the second regressions we also include a number of individual control variables (as discussed in Subsection **??**). We estimated marginal effects for all logit regressions. Table 1 reports the marginal effects of the score variables for Game 9, while Table 2 reports the marginal effects of the score variables for Game 10.

B.3 Interpreting observed performance in Game 9 and Game 10

The proposed framework based on the concepts of rationality and mentalization suggests an explanation of the observed performance in Game 9 and Game 10 by the players in our sample.

Game 9 represents a non-trivial strategic situation where the first player who has to move, Tizio, has an option to commit to a greater loss in case of retreat. Since such action is observed by the second player, Caio, the latter can attach a meaning to the action. This in turn allows Tizio to use the action to convey information, namely that he will not be willing to retreat. Note that the success of such implicit communication relies on Caio correctly understanding Tizio's strategic situation and, crucially, in considering Tizio capable of understanding it too. The fact that a higher *Riter* predicts more likely success in all scores of Game 9 is consistent with this interpretation. This is reinforced by the fact that *Mskil* predicts well Caio's performance when he observes Tizio to commit to a greater loss in case of retreat (g92), while this

Marginal effects for scores in GAME 9

VARIABLE	(1) g91	(2) g91	(3) g92	(4) g92	(5) g93	(6) g93	(7) g94	(8) g94
Mpref	0.0431	0.0350	0.0092	-0.0034	0.0062	-0.0128	-0.0002	-0.0007
	(0.0304)	(0.0297)	(0.0345)	(0.0347)	(0.0361)	(0.0356)	(0.0332)	(0.0328)
Mskil	0.0188	0.0068	0.0735*	0.0723*	-0.0360	-0.0580	0.0132	-0.0005
	(0.0319)	(0.0319)	(0.0342)	(0.0347)	(0.0365)	(0.0355)	(0.0350)	(0.0347)
Ropti	0.0139	0.0367	0.0574	0.0665^{+}	0.0302	0.0517	-0.0082	0.0081
	(0.0307)	(0.0325)	(0.0354)	(0.0368)	(0.0348)	(0.0344)	(0.0334)	(0.0349)
Riter	0.0571^{+}	0.0440	0.1435**	0.1367**	0.0674^{+}	0.0454	0.1091**	0.0931**
	(0.0294)	(0.0280)	(0.0309)	(0.0312)	(0.0355)	(0.0350)	(0.0321)	(0.0316)
male		0.1824**		0.0997		0.2175**		0.1034
		(0.0628)		(0.0687)		(0.0654)		(0.0675)
monthly income		0.0121		-0.0183		0.0457		0.0136
		(0.0355)		(0.0418)		(0.0416)		(0.0405)
age		0.0004		-0.0076		-0.0178		-0.0098
		(0.0095)		(0.0103)		(0.0112)		(0.0117)
completed college		-0.0908		0.0341		-0.0146		-0.0745
		(0.0657)		(0.0761)		(0.0780)		(0.0757)
father college		-0.0350		0.0481		-0.0034		0.1270
		(0.0771)		(0.0914)		(0.0930)		(0.0842)
mother college		0.0596		-0.0224		-0.0162		0.0580
		(0.0772)		(0.0956)		(0.0975)		(0.0882)
Observations	187	186	187	186	187	186	187	186
Individual controls	No	Yes	No	Yes	No	Yes	No	Yes

Table 1: Marginal effects for logit regressions. Dependent variables are games scores for Game 9 (indicated below the number of the regression). Statistical significance is denoted by ** if p-value <0.01, * p-value <0.05, and $^{+}$ if p-value < 0.1. Standard errors are in parentheses. Estimated coefficients are omitted.

Marginal effects for scores in GAME 10						
	(1)	(2)	(3)	(4)	(5)	(6)
VARIABLES	g101	g101	g102	g102	g103	g103
Mpref	0.0748*	0.0702*	0.0263	0.0349	-0.0064	-0.0161
	(0.0308)	(0.0306)	(0.0352)	(0.0353)	(0.0323)	(0.0312)
Mskil	-0.0163	-0.0199	-0.0194	-0.0168	0.0361	0.0318
	(0.0299)	(0.0300)	(0.0362)	(0.0365)	(0.0339)	(0.0331)
Ropti	-0.0298	-0.0370	0.0023	-0.0058	0.0388	0.0701^{+}
	(0.0250)	(0.0258)	(0.0354)	(0.0362)	(0.0366)	(0.0386)
Riter	0.0420	0.0417	0.0876*	0.0955**	-0.0004	-0.0040
	(0.0294)	(0.0303)	(0.0346)	(0.0354)	(0.0322)	(0.0305)
male		-0.0129		-0.1162		0.1457*
		(0.0602)		(0.0720)		(0.0641)
monthly income		0.0395		0.0281		-0.0784*
		(0.0346)		(0.0429)		(0.0380)
age		0.0001		0.0062		-0.0083
		(0.0090)		(0.0097)		(0.0102)
completed college		-0.0551		-0.0667		0.0205
		(0.0655)		(0.0765)		(0.0698)
father completed college		-0.0097		-0.0756		-0.1743^{+}
		(0.0780)		(0.0955)		(0.0898)
mother completed college		-0.1139		0.0188		0.1883*
		(0.0842)		(0.1003)		(0.0886)
Observations	187	186	187	186	187	186
Individual controls	No	Yes	No	Yes	No	Yes

Table 2: Marginal effects for logit regressions. Dependent variables are games scores for Game 10 (indicated below the number of the regression). Statistical significance is denoted by ** if p-value <0.1, * p-value <0.05, and $^{+}$ if p-value < 0.01. Standard errors are in parentheses. Estimated coefficients are omitted.

is not so when such action is not observed (g93). At the same time, both *Mpref* and *Mskil* seem to be somewhat relevant for Tizio's performance against Caio (g91, see conditional means in Figure 1), suggesting that knowing the opponent's preference may be useful, reasonably in order to estimate the likelihood that Caio prefers to attack anyway because he likes it (in which case the implicit communication is useless), as well as knowing opponent's skill, reasonably because Caio needs to understand what are Tizio's options to give them a meaning. At the same time, *Ropti* seems to play only some role in Caio's performance when he observes Tizio to commit to a greater loss in case of retreat (g92), suggesting that Caio has to make some more calculations than in other parts of Game 9, possibly in order to realize that Tizio is incurring in greater loss if he retreats.

Game 10 represents a different non-trivial strategic situation where there is a cake that grows at each stage of the game. In every stage a player has, in turn, the chance to terminate the game and take the bigger slice of the cake, leaving the remaining part to the other player. In a sense this is a game of trust since it is always better not to terminate the game if one expects that the other player will do the same in the following stage. However, each player has an incentive to be untrustworthy since only the player who terminates the game can get the largest slice of the cake. In this game *Riter* still seems to play an important role, but to

a lesser extent than in Game 9. In particular, *Riter* seems to be more relevant for the player who is the second to move, Caio, reasonably because he has to give an interpretation of why the first player, Tizio, did not terminate the game immediately getting the largest slice of the cake (indeed, Caio has the possibility to act only if Tizio does not terminate the game in the first stage). *Mskil* seems to have almost no role while *Ropti* seems to be somewhat relevant when Tizio plays against an automaton caring only about its stake (g103). Instead, *Mpref* is quite relevant for the performance of Tizio against the other participants to the test playing the role of Caio (g101), presumably because Tizio has to figure out whether Caio will terminate the game in the stage immediately after or will let game continue (which is the only case in which Tizio can benefit from not terminating immediately the game). This possibility may depend on the preference of Caio regarding a more equal split of the cake or a preference for social efficiency (i.e., obtaining a greater cake no matter who takes the largest slice), which might motivate Caio not to terminate the game as soon as he has the chance to do so.

These interpretations, which are consistent with standard game-theoretical accounts of strategic behavior based on forward induction (Govindan and Wilson, 2009) in Game 9 and social preferences (Levitt and List, 2007) in Game 10, point to the possibility that our analysis helps in dissecting and explaining strategic behavior.

B.4 Relation with other psychological measures

One source of individual information is provided by the pre-test questions regarding risk preferences, time preferences, fairness, and cognitive skills; in addition, we administered to the same sample of subjects a number of other tests to explore correlations and provide a basis for interpreting the data (see Subsection 2.2).

Table 3 reports the pairwise correlations. *Mpref* has a statistically significant correlation only with a measure of fairness (Reject 95-5) and with two personality trait, Conscientiousness and Neuroticism, and in all cases is relatively small and negative. *Ropti* shows no statistically significant correlations. *Mskil* and *Ropti* have a statistically significant correlation with all variables incorporating some measure of reasoning ability (pre-test 3-item IQ, Raven score, CRT, Rational attitude). This is not surprising since both factors also necessarily incorporate some measure of reasoning ability (to understand skills for *Mskil* and to iterate strategic reasoning for *Riter*). This, however, is in stark contrast with the absence of correlation for the factor *Ropti*, suggesting that basic optimization is not captured at all by these measures of reasoning ability. It is also interesting that the 6-item CRT measure shows a strong correlation (.41) with *Riter*, substantially larger than any other measure of reasoning ability. This is in line with our understanding of strategic thinking: in order to realize that the environment is strategic, and therefore dependent on others' decisions, and in order to take this properly into account, a player has often to reflect and deliberate with substantial cognitive effort, avoiding too fast and cognitively cheap decision-making. Furthermore, both *Mskil* and *Riter* have a negative and statistically significant correlation with Experiential attitude. This is also not surprising, indicating that these factors are less likely to be associated with a more experiential approach to judgment, as opposed to a more rational one. Finally, *Riter* has a negative and statistically significant correlation with Extroversion, but we do not have a straightforward interpretation for this. Perhaps more interesting is the fact that extroverts do not seem to have higher scores of *Mpref* and *Mskil* (non-significant correlation of .05 and .06, respectively), as one might expect due to a larger exposure to interactions with peers. One possible reason is that extroverts may not learn more about others, even if they tend to interact with more people,

	Mpref	Mskil	Ropti	Riter
Less money, less risk	0.0224	-0.0742	-0.0087	-0.1145
	(0.7611)	(0.3126)	(0.9058)	(0.1187)
Less money, all now	0.0146	-0.0740	0.0644	-0.0942
	(0.8431)	(0.3152)	(0.3822)	(0.2010)
Reject 95-5	-0.2507**	-0.0102	0.0039	-0.0422
	(0.0006)	(0.8896)	(0.9578)	(0.5677)
3-item IQ	0.1197	0.2235**	-0.0140	0.3115**
	(0.1029)	(0.0021)	(0.8496)	(0.0000)
Raven APM score	-0.0411	0.1923**	0.0542	0.2758**
	(0.5765)	(0.0084)	(0.4621)	(0.0001)
CRT6	0.0957	0.1933**	0.0363	0.4132**
	(0.1926)	(0.0080)	(0.6228)	(0.0000)
Rational attitude	0.0921	0.1490*	0.0511	0.2590**
	(0.2100)	(0.0419)	(0.4888)	(0.0003)
Experiential attitude	-0.0742	-0.1833*	-0.0983	-0.1588*
	(0.3130)	(0.0120)	(0.1819)	(0.0299)
Extroversion	0.0509	0.0655	0.0197	-0.1877**
	(0.4891)	(0.3728)	(0.7895)	(0.0101)
Agreeablenness	-0.0619	0.1066	-0.0418	0.0104
	(0.4000)	(0.1465)	(0.5710)	(0.8878)
Conscientiousness	-0.2242**	-0.0269	0.1278	-0.1112
	(0.0020)	(0.7147)	(0.0821)	(0.1299)
Neuroticism	-0.1730*	0.0162	0.0180	-0.0876
	(0.0179)	(0.8258)	(0.8077)	(0.2333)
Openess	0.0832	-0.0926	-0.0006	-0.0361
	(0.2579)	(0.2075)	(0.9934)	(0.6233)

Table 3: Pairwise correlations of the four components extracted with other variables recorded (p-values in parentheses). "Less money, less risk" is a binary variable (0 or 1) coding the choice between two hypothetical lotteries (1 is for the less risky but with lower expected value). "Less money, all now" is a binary variable (0 or 1) coding the choice between two streams of hypothetical payments (1 is for smaller but without delay). "Reject 95-5" is a binary variable coding the choice to accept (0) or reject (1) an unfair split of 100 euro, where in case of rejection everybody gets 0. "3-item IQ" is the number of correct answers (0 to 3) to cognitive skill questions in the pre-test. "Raven APM score" is the number of correct answers in the Raven's Advanced Progressive Matrices test. CRT6 is the score in the cognitive reflection test with 6 items (Primi et al., 2016). Rational attitude and Experiential attitude are measured with the REI40 (Pacini and Epstein, 1999). The remaining variables are the BIG5 personality traits measured with the 46 items inventory (John et al., 1991).

when this entails less frequent interactions with the same individuals.

Overall, these correlation figures suggest that the four factors extracted capture different dimensions from those captured by standard measures of preferences, reasoning and personality, with the only exception perhaps of the CRT which correlates strongly with iterative rationality.

References

Govindan, S. and R. Wilson (2009). On forward induction. *Econometrica 77*(1), 1–28.

John, O. P., E. M. Donahue, and R. L. Kentle (1991). The big-five inventory-versions 4a and 54. Technical report, Berkeley, CA: University of California, Berkeley, Institute of Personality and Social Research.

Levitt, S. D. and J. A. List (2007). What do laboratory experiments measuring social preferences reveal about the real world? *Journal of Economic Perspectives 21*(2), 153–174.

Pacini, R. and S. Epstein (1999). The relation of rational and experiential information processing styles to personality, basic beliefs, and the ratio-bias phenomenon. *Journal of Personality and Social Psychology 76*(6), 972.

Primi, C., K. Morsanyi, F. Chiesi, M. A. Donati, and J. Hamilton (2016). The development and testing of a new version of the cognitive reflection test applying item response theory (irt). *Journal of Behavioral Decision Making 29*(5), 453–469.
