# Supplementary material for: Assessing Actual Strategic Behavior to Construct a Measure of Strategic Ability
Source: Front Psychol. 2019 Jan 18;9:2750. doi: 10.3389/fpsyg.2018.02750 (PMC6345706; doi:10.3389/fpsyg.2018.02750)
Supplement: Supplementary Data Sheet S1 — SQ Test — Translated English version. [file Data_Sheet_1.PDF]

English version of instructions and test

Disclaimer The original Strategic Quotient Test is written in Italian. The Italian version has been repeatedly checked for ambiguous parts or points in which a subset of subjects consistently gave a different interpretation to the text. The version of the test used in the presented research has been deemed free of such parts, as subjects did not report any. Here we report an english-translated version for reference. The english version has not been tested in any way, but serves the only purpose of presenting the games to the reader. As such, it may not be taken as an english version of the Strategic Quotient Test

PRE-TEST

Before starting the SQ, you need to answer question D1-D5 in the following. These questions are needed for setting some parameters of the SQ test. Your answers to this pre-test do **not affect** your SQ score. **Take all the time you need to provide answers**. When you are done, turn the page and read the instructions of the SQ.

D1. Select the lottery you would prefer to participate in:

[ ] lottery "A": you win 400 € with a probability of 50%, while you win 0 € with the probability of 50% (expected value 200 €)

[ ] lottery "B": you win 100 € with a probability of 99%, while you win 200 € with the probability of 1% (expected value 101 €)

D2. Select what you would prefer between:

[ ] choice "A": to receive 50 € immediately and 500 in a year (total 550 €)

[ ] choice "B": to receive 350 immediately (total 350 €)

D3. Select how you would behave in the following situation:

A person has the possibility of being given a sum of 100 €, but only if s/he shares a part of it with you and that you agree on the proposed split. This person offers a split of 95 € for her/him and 5 € for you.

(D3.1) What do you do?

[ ] option "ACCEPT": you take 5 € and that person takes 95 €

[ ] option "REJECT": you and the other person both take 0 €

(D3.2) In general, what would it be the minimum amount of € for you that would convince you to ACCEPT the split made by this person? …………………€

D4. Select the correct answer for each of the following 3 questions (there is only one correct answer for each):

(D4.1) Select the number that follows in the correct numerical series 8, 24, 12, 36, 18, 54:

168 [ ]   102 [ ]   27 [ ]   56/3 [ ]   18 [ ]

(D4.2) Consider the statement "When all hospitals are full, then no sick person can be cured" and select which of the following sentences falsifies it:

"There is an empty hospital" [ ]

"There is a sick person that can be cured" [ ]

"All hospitals are full, but a sick person was cured" [ ]

"Not all hospitals are full and a new sick person has was cured" [ ]

"If the hospitals are empty, then no sick person can be cured" [ ]

(D4.3) Select the figure to delete among the following pictures:

A [ ]

B [ ]

C [ ]

D [ ]

E [ ]

D5. Honestly, what would be the maximum price you would pay for participating in a test that would measure your strategic ability in a reliable manner?

Keep in mind that obviously you won't pay this test.

Maximum price: ……………………..€

GENERAL INSTRUCTIONS FOR THE SQ TEST

READ CAREFULLY!

Read this instructions carefully, **taking all the time you need**.

When you think you correctly understood the instructions and you completed the previous pre-test, **call an experimenter and tell her/him you finished**. If you have doubts of any kind, please ask her/him.

The SQ test will be given to you and, from that moment, you have **30 minutes** to take the test.

HOW TO WIN THE GAMES IN THE TEST?

In the games of this test **there are no scores**, but there are **goals to reach**. To do well in this test, remember that **in each game you need to make the choices that, depending on the circumstances, you think allow you to win (or get the maximum prize)**. It is important that you keep in mind that in many cases a win in a game is determined on the basis of the choices that **all participants to the test make**, and as such even your *Strategic Quotient* depends on those choices. Keep in mind also that all the information needed in order to win a game are fully described in the text.

PLAYING A GAME TWICE IN THE SHOES OF TWO DIFFERENT PLAYERS

In some games you'll find two players, Tizio and Caio. In first place, you'll be required to make your choices as if you were Tizio, and in a second moment you'll be required to make your choices as if you were Caio. **All the choices you'll make in the shoes of each player** need to be those choices that you think **allow that player to win the game (or the maximum prize)**: you need to make Tizio win the maximum prize when you are required to play as Tizio, while you need to make Caio win when you are required to play as Caio. In both cases, **the choices of your opponent** (Caio when you are playing as Tizio, and Tizio when you are playing as Caio) **will be determined on the basis of the choices of the other participants to this test.**

> *For example, in a game in which Tizio needs to choose between A and B and Caio needs to choose between C and D, your score as Tizio is determined using your choice in the shoes of Tizio (A or B) against an "average" choice Caio made determined from the percentage of participants to this test that, while playing as Caio, choose C and the percentage of those who choose D (if 50% of the participants choose C and 50% choose D then the choice of Caio will be C with a probability of 50% and D with a probability of 50%).*

In some cases you'll play against a **specific sub-group of participants to this test**. In those cases you'll find all the needed details in the text of the game.

MISTAKES

To rectify an answer you already gave after changing your mind, you only need to write "no" near the wrong answer and proceed answering as usual.

CALCULATING THE *STRATEGIC QUOTIENT*

For the total SQ score, **each game counts more or less as any other game**.

Game 1. Write the percentage of participants that in your opinion made these choices in the questions D1, D2, D3:

Lottery “A”: ……….%

Choice “A”: ……….%

Choice “ACCEPT”: ……….%

Write the percentage of participants that in your opinion answered correctly to:

Question (D4.1): ……….%

Question (D4.2): ……….%

Question (D4.3): ……….%

Write how much was the average of the declared maximum price in question D5: ……….€

NOTE: the score you’ll receive will be as higher as you’ll get closer to the real choices made by the participants to this test.

Game 2. In this game you need to guess what participants to this test think about other participants. Specifically, you need to guess the average responses to the questions of Game 1 or, in other words, what other participants to this test think about other participants’ answers to questions D1, D2, D3, D4, D5.

On average, participants to this test think that:

the ……….% of participants choose Lottery “A” in question D1

the ……….% of participants choose Choice “A” in question D2

the ……….% of participants choose Choice “ACCEPT” in question D3

the ……….% of participants answered question D4.1 correctly

the ……….% of participants answered question D4.2 correctly

the ……….% of participants answered question D4.3 correctly

the average declared price in question D5 was ……….€

NOTE: the score you’ll receive will be as higher as you’ll get closer to the actual average of the answers in Game 1 made by the participants to this test.

Game 3. There are two players, Tizio and Caio. Tizio chooses secretly a letter among: A, B, C, D, E, F. Tizio wins a prize if he chooses E, while he wins nothing if he chooses other letters. Caio chooses a letter as well, among the following: A, B, C, D, E, F. If Caio chooses the same letter as Tizio, Caio wins as well, otherwise he gets nothing.

Keep in mind that both players know these instructions.

Suppose you are Tizio, and **suppose that Caio’s choices are determined by the choices of the other participants to this test when they play as Caio**; what do you choose?

A [ ]   B [ ]   C [ ]   D [ ]   E [ ]   F [ ]

Now suppose you are Caio, and **suppose that Tizio's choices are determined by the choices of the other participants to this test when they play as Tizio**; what do you choose?

A [ ] B [ ] C [ ] D [ ] E [ ] F [ ]

Now suppose you are still Caio, and **suppose that Tizio's choices are made by an automaton that always chooses in order to win the prize**; what do you choose?

A [ ] B [ ] C [ ] D [ ] E [ ] F [ ]

Game 4. Tizio and Caio face a dangerous duel with peculiar rules.

Tizio chooses a weapon, then Caio uses the chosen weapon to hit Tizio. **If Caio hits Tizio, Caio wins the duel**. **If he misses, Tizio wins**.

Tizio chooses between a gun, a fencing sword, a bow and an iron club. Caio takes the chosen weapon and strikes one blow, after deciding whether "aiming for the head" or "aiming for the body". The chance that the blow hits depends on the weapon chosen by Tizio and on what Caio decided to aim for as follows:

- with the gun, Caio hits with a probability of 60% if aiming for the body and 30% if aiming for the head

- with the fencing sword, Caio hits with a probability of 50% if he aims for the body as well as if he aims for the head

- with the bow, Caio hits with a probability of 10% if aiming for the body and 55% if aiming for the head

- with the iron club, Caio hits with a probability of 25% if aiming for the body and 60% if aiming for the head

Suppose you are Tizio, and **suppose that the Caio's choices are determined by the choices of the participants to this test when playing as Caio**; what do you choose?

gun [ ] fencing sword [ ] bow [ ] iron club [ ]

Now suppose you are Caio, what do you choose? [answer for each possible Tizio's choice]

If Tizio chooses the gun, you aim for: the head [ ] the body [ ]

If Tizio chooses the fencing sword, you aim for: the head [ ] the body [ ]

If Tizio chooses the bow, you aim for: the head [ ] the body [ ]

If Tizio chooses the iron club, you aim for: the head [ ] the body [ ]

Now suppose you are Tizio again, and **suppose that Caio's choices are made by an automaton that chooses trying to find the maximum probability for winning**; what do you choose?

gun [ ] fencing sword [ ] bow [ ] iron club [ ]

Game 5. Choose a color among the following, with **the goal of choosing the one that will be the chosen the most by the other participants to this test**.

brown [ ] red [ ] blue [ ] yellow[ ] green [ ] grey [ ]

With the same **goal of choosing the color that will be chosen the most by the other participants to this test**, choose a number among the following after reading this sentence: “Each year is made of twelve months”.

18 [ ] 9 [ ] 24 [ ] 12 [ ] 5 [ ] 31 [ ]

Game 6. The goal of this game is to choose a number as close as possible to two thirds (around 66%) of the **mean of the numbers chosen in this game by the other participants to this test**. (For example, if the mean of the chosen numbers is 30, you obtain the highest possible score by choosing 20, that is two thirds of 30). Choose a whole number between 1 and 90: ……………

Now choose a number as close as possible to two thirds (around 66%) of the mean of the number chosen by the other participants to this test who **correctly answered all questions D4.1**, **D4.2**, **D4.3**. Choose a whole number between 1 and 90: ……………

Finally, choose a number as close as possible to two thirds (around 66%) of the number chosen by **an automaton**. This automaton **makes its choice after having seen the number you choose** and always chooses the number closer to 2/3 of yours. Choose a whole number between 1 and 90: ……………

Game 7. Consider a situation in which Tizio and Caio have to split 100 € with the following rules:

> Tizio offers how to split 100 €; then, Caio decides whether to accept or reject. If the split is accepted by Caio, the sum is divided as offered. If the split is rejected by Caio, all the money gets lost, and both Tizio and Caio receive 0 €.

Suppose you are Tizio, and **suppose that Caio is an automaton that chooses if accepting or rejecting the offered split on the basis of what allows it to gain the maximum amount of money**. You have to offer the split: how much money do you offer to Caio in the split?

(Remember: your goal is to keep as much money for yourself as possible.)

Write a number between 0 and 100: ………….

Suppose you are Tizio, and **suppose that Caio’s choices are determined by the choices of the other participants to question D3.2**. How much money do you offer to Caio in the split?

(Remember: your goal is to keep as much money for yourself as possible.)

Write a number between 0 and 100: ………….

Game 8. Suppose you want to commercialize a test that reliably measures the strategic skills of individuals. Specifically, you need to choose the price for a single administration of the test with the aim of **maximizing your revenue**. Your revenue will be calculated using the **actual answers given to question D5** by the other participants to this test: if you choose a certain prize, you’ll sell the test at that price but only to those who declared to be willing to pay at least that price for the test.

(Since you are interested in your revenue, **do not consider** any possible development or distribution cost.)

Price for a single administration: ……………€

Game 9. Two armies are deployed one in front of the other. At the head of one army there is commander Tizio, while at the head of the other there is commander Caio.

Both Tizio and Caio can choose either to charge or retreat. These choices will be made simultaneously: each commander will choose without knowing what the other will choose.

Each army can **win**, **draw**, **lose** or **be annihilated** (winning is preferred to drawing, which in turn is preferred to losing, which in turn is preferred to being annihilated).

If both armies charge, then a massacre will occur: the battle will end with no winners and no survivor will remain; in this case, both armies will **be annihilated**. Instead, if one army charges and the other retreats, the charging army will **win** and the retreating one will **lose**. If both armies retreat, they both **draw**.

Additionally, behind Tizio's army there is a river that can be crossed only through a bridge. Before the battle starts, Tizio can decide to demolish the bridge. In the case the bridge is demolished, if Tizio's army retreats all soldiers will drown in the water and Tizio's army will be **annihilated**. Caio has no such possibility of demolishing any bridge, and will always be able to retreat without having his army being annihilated.

The following table sums up what can happen:

	**Tizio charges**	**Tizio retreats**
**Caio charges**	*The bridge is untouched:* both armies are annihilated<br>*The bridge is demolished:* both armies are annihilated	*The bridge is untouched:* Tizio loses, Caio wins<br>*The bridge is demolished:* Caio wins, Tizio's army is annihilated
**Caio retreats**	*The bridge is untouched:* Tizio wins, Caio loses<br>*The bridge is untouched:* Tizio wins, Caio loses	*The bridge is untouched:* both draw<br>*The bridge is demolished:* both draw

Hence:

- Charging is always the best choice if the other army retreats.

- If Tizio doesn't demolish the bridge, for him retreating is the best choice if Caio charges.

- If Tizio demolishes the bridge, for him retreating and charging have the same outcome (being annihilated) if Caio charges.

- For Caio retreating is always the best choice if Tizio charges.

Keep in mind that, before deciding what to do, **Caio observes if Tizio has demolished the bridge or not**.

Suppose you are Tizio, and **suppose that Caio's choices are determined by the choices of the participants to this test when playing as Caio**. What do you order to your army?

Demolish the bridge? Yes [ ] No [ ]

Then what do you order?: Charge [ ] Retreat [ ]

Suppose you are Caio, and **suppose that the Tizio's choices are determined by the choices of the participants to this test when playing as Tizio**. What do you order to your army?

If Tizio demolished the bridge, what to do? Charge [ ] Retreat [ ]

If Tizio didn't demolish the bridge, what to do? Charge [ ] Retreat [ ]

Suppose you are again Tizio, and **suppose that Caio's choices are determined by the choices of the participants to this test who answered correctly questions D4.1, D4.2, D4.3**. What do you order to your army?

Demolish the bridge? Yes [ ] No [ ]

Then what do you order: Charge [ ] Retreat [ ]

Game 10. Consider the following situation in which Tizio and Caio participate in the following step-by-step game:

Step 1:

**TIZIO** chooses if:	**ending** the game with: 200€ to Tizio, 0€ to Caio	or	**continue** the game and go to step 2

Step 2:

**CAIO** chooses if:	**ending** the game with: 100€ to Tizio, 300€ to Caio	or	**continue** the game and go to step 3

Step 3:

**TIZIO** chooses if:	**ending** the game with: 400€ to Tizio, 200€ to Caio	or	**continue** the game and go to step 4

Step 4:

**CAIO** chooses if:	**ending** the game with: 300€ to Tizio, 500€ to Caio	or	**continue** the game and go to step 5

Step 5:

**TIZIO** chooses if:	**ending** the game with: 600€ to Tizio, 400€ to Caio	or	**continue** the game and go to step 6

Step 6:

**CAIO** chooses if:	**ending** the game with: 500€ to Tizio, 700€ to Caio	or	**ending** the game with: 600€ to Tizio, 600€ to Caio

Suppose you are Tizio, and **suppose that Caio's choices are determined by the choices of the participants to this test when playing as Caio**. Suppose you need to take all your decisions in advance without knowing what Caio will do. **Keep in mind that both players know these instructions**.

(Remember: your goal is to obtain at the end of the game the highest amount of money for yourself.)

What do you choose?

Ending the game at step 1 [ ] Ending the game at step 3 [ ]

Ending the game at step 5 [ ] Always continue [ ]

Suppose you are Caio, and **suppose that Tizio's choices are determined by the choices of the participants to this test when playing as Tizio**. Suppose you need to take all your decisions in advance without knowing what Tizio will do.

(Remember: your goal is to obtain at the end of the game the highest amount of money for yourself.)

What do you choose?

Ending the game at step 2 [ ] Ending the game at step 4 [ ]

Ending the game at step 6 with 500€ to Tizio, 700 € to Caio [ ]

Ending the game at step 6 with 600€ to Tizio, 600 € to Caio [ ]

Suppose you are Tizio again, and **now suppose that Caio's choices are made by an automaton that wants to gain the highest amount of money for itself and can predict your choice**. (Remember: your goal is to obtain at the end of the game the highest amount of money for yourself.)

What do you choose?

Ending the game at step 1 [ ] Ending the game at step 3 [ ]

Ending the game at step 5 [ ] Always continue [ ]
