# Supplementary material for: Assessing Actual Strategic Behavior to Construct a Measure of Strategic Ability
Source: Front Psychol. 2019 Jan 18;9:2750. doi: 10.3389/fpsyg.2018.02750 (PMC6345706; doi:10.3389/fpsyg.2018.02750)
Supplement: Supplementary Data Sheet S2 — SQ Test — Original Italian version. [file Data_Sheet_2.PDF]

PRE-TEST

Prima del test QS vero e proprio dovrai rispondere alle domande D1-D5 che trovi di seguito e che servono per determinare alcuni parametri del test del QS. Le tue risposte a questo pre-test **non influenzano** il tuo punteggio di QS. **Prenditi tutto il tempo che ti serve per rispondere**. Quando hai fatto, volta pagina e leggi le istruzioni del test QS vero e proprio.

D1. Indica a quale lotteria preferiresti partecipare: ✎

□ lotteria "A": al 50% vinci 400 euro mentre al 50% vinci 0 euro (valore atteso 200 euro)
□ lotteria "B": al 99% vinci 100 euro mentre al 1% vinci 200 euro (valore atteso 101 euro)

D2. Indica cosa preferiresti: ✎

□ scelta "A": ricevere 50 euro subito e 500 euro tra 1 anno (totale euro 550)
□ scelta "B": ricevere 350 euro subito (totale euro 350)

D3. Indica quale sarebbe il tuo comportamento nella seguente situazione:
Una persona ha la possibilità di ottenere 100 euro, ma solo a patto che ne divida una parte con te e che tu dia il tuo consenso alla divisione proposta. Questa persona ti propone di tenere per sé 95 euro e di lasciare a te 5 euro.

(D3.1) Cosa fai? ✎

□ opzione "ACCETTO": ti prendi i 5 euro e questa persona ne prende 95
□ opzione "RIFIUTO": prendi 0 euro sia tu che l'altra persona

(D3.2) In generale, qual è l'ammontare minimo di euro lasciati a te che ti convincerebbe ad ACCETTARE la proposta fatta da questa persona? ✎ …….. euro

---

D4. Indica la risposta esatta (che è unica) per ciascuno dei seguenti 3 quesiti:

(D4.1) Indica quale numero segue nella sequenza numerica 8, 24, 12, 36, 18, 54: ✎
168 □ 102 □ 27 □ 56/3 □ 18 □

(D4.2) Considera l'affermazione "Quando tutti gli ospedali sono pieni, allora nessun nuovo malato può essere curato" ed indica quale tra le seguenti frasi la smentisce: ✎
"C'è un ospedale vuoto" □ "C'è un malato che può essere curato" □
"Gli ospedali sono tutti pieni, ma un nuovo malato è stato curato" □
"Gli ospedali non sono tutti pieni e un nuovo malato è stato curato" □
"Se gli ospedali sono vuoti, allora nessun malato può essere curato" □

(D4.3) Individua la figura da eliminare tra le seguenti figure: ✎

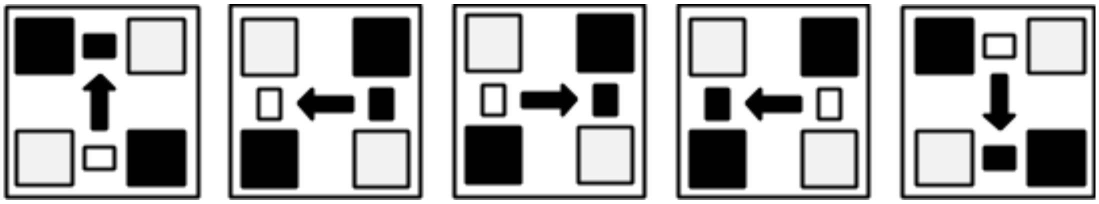

A □ B □ C □ D □ E □

D5. In sincerità, quale è il massimo prezzo che saresti disposto a pagare per partecipare ad un test che misuri la tua capacità strategica in maniera attendibile?
Tieni presente che ovviamente non ti verrà chiesto di pagare questo test.

Prezzo massimo: ✎ …………. euro [tra 0 e 100]

ISTRUZIONI GENERALI DEL TEST QS

LEGGERE CON ATTENZIONE!

Leggi con attenzione queste istruzioni, **prendendoti tutto il tempo che ti serve**.
Quando pensi di aver capito correttamente le istruzioni e di aver compilato correttamente il pre-test precedente, **chiama un somministratore e avvertilo che hai terminato**.
Se hai dei dubbi, di qualunque genere, ponigli tutte le domande che reputi opportune.
Ti verrà consegnato il test del QS; da quel momento, avrai **30 minuti in tutto** per svolgerlo.

COME VINCERE NEI GIOCHI DEL TEST?
Nei giochi di questo test **non sono forniti punteggi**, ma **obiettivi da raggiungere**. Per fare bene questo test ricorda che **in ogni gioco dovrai cercare di fare le scelte che, a seconda dei casi, pensi ti garantiscano la vittoria (o il massimo premio in palio)**. E' importante che tu tenga a mente che in molti casi la vittoria in un gioco dipende dalle scelte che fanno **tutti i partecipanti al test**, e dunque anche il tuo *Quoziente Strategico* dipende da tali scelte. Tieni anche presente che tutte le informazioni necessarie a vincere un gioco sono indicate nel testo.

GIOCARE UN GIOCO DUE VOLTE NEI PANNI DI DUE DIVERSI GIOCATORI
In alcuni giochi troverai due giocatori, Tizio e Caio. Prima ti verrà chiesto di fare le tue scelte supponendo di essere Tizio e, successivamente, ti verrà chiesto di fare le tue scelte supponendo di essere Caio. **Tutte le scelte** che farai dovranno essere quelle che pensi **garantiscano la vittoria (o il massimo premio in palio) al giocatore che ti è stato chiesto di interpretare in quel momento** (cioè devi cercare di far vincere il massimo premio possibile a Tizio quando ti viene chiesto di supporre di essere Tizio, a Caio quando ti viene chiesto di supporre di essere Caio). In entrambi i casi **le scelte del tuo avversario** (Caio quando tu giochi come Tizio e Tizio quando tu giochi come Caio) **saranno quelle fatte dagli altri partecipanti a questo test.**

> *Ad esempio, in un gioco dove Tizio deve scegliere tra A e B e Caio tra C e D, il tuo punteggio come Tizio è calcolato usando la tua scelta nel ruolo Tizio (A o B) contro una scelta "media" per Caio fatta dalla percentuale dei partecipanti al test nel ruolo di Caio che hanno scelto C e dalla percentuale di quelli che hanno scelto D (se metà dei partecipanti ha scelto C e metà ha scelto D allora la scelta di Caio sarà con probabilità 50% la scelta C e 50% la scelta D).*

In alcuni casi giocherai contro un **particolare sotto-gruppo di partecipanti al test**. In tali casi, troverai i dettagli relativi nel testo del gioco.

RISPOSTE DATE ERRONEAMENTE
Per correggere una risposta già data ma che si ritiene sbagliata, basta scrivere "no" a fianco di quella errata e dare la risposta corretta normalmente.

CALCOLO DEL *QUOZIENTE STRATEGICO*
Per il punteggio totale del test **ciascun gioco conta circa allo stesso modo**.

Gioco 1. Indica, secondo te, quale percentuale di partecipanti a questo test ha fatto le seguenti scelte nelle domande D1, D2, D3:

la lotteria “A”: ✎ …..%
la scelta “A”: ✎ …..%
l’opzione “ACCETTO” (D3.1): ✎ …..%

Indica, secondo te, quale percentuale di partecipanti ha risposto **correttamente** a:

domanda (D4.1): ✎ ….. %
domanda (D4.2): ✎ ….. %
domanda (D4.3): ✎ ….. %

Indica, secondo te, quale è il prezzo medio dichiarato nella domanda D5: ✎ …... euro

NOTA: il punteggio che otterrai sarà tanto più grande quanto più ti avvicinerai alle scelte realmente effettuate dai partecipanti a questo test.

Gioco 2. In questo gioco devi indovinare cosa i partecipanti a questo test pensano degli altri partecipanti. In particolare, devi indovinare quali sono state, in media, le risposte al Gioco 1; in altre parole, devi indovinare cosa i partecipanti al test pensano che gli altri partecipanti abbiano risposto alle domande D1, D2, D3, D4, D5.

In media i partecipanti a questo test pensano che:

il ✎ ….. % dei partecipanti abbia scelto la lotteria A nella domanda D1
il ✎ ….. % dei partecipanti abbia scelto la scelta A nella domanda D2
il ✎ ….. % dei partecipanti abbia scelto l’opzione ACCETTO nella domanda D3
il ✎ ….. % dei partecipanti abbia risposto correttamente alla domanda D4.1
il ✎ ….. % dei partecipanti abbia risposto correttamente alla domanda D4.2
il ✎ ….. % dei partecipanti abbia risposto correttamente alla domanda D4.3
✎ …….. euro sia il prezzo massimo dichiarato mediamente alla domanda D5

NOTA: il punteggio che otterrai sarà tanto più grande quanto più ti avvicinerai alla media effettiva delle dichiarazioni fatte dai partecipanti nel Gioco 1.

Gioco 3. Ci sono due giocatori, Tizio e Caio. Tizio sceglie in segreto una lettera tra: A, B, C, D, E, F. Tizio vince un premio se sceglie E, mentre non vince niente se sceglie un’altra lettera.
Anche Caio deve scegliere una lettera tra: A, B, C, D, E, F. Se sceglie la stessa lettera scelta da Tizio ottiene un premio anche lui, altrimenti non ottiene niente.

Tieni presente che entrambi i giocatori conoscono queste istruzioni.

Supponi di essere Tizio, e **supponi che le scelte di Caio siano determinate dalle scelte dei partecipanti a questo test nei panni di Caio**; cosa scegli?

✎ A □ B □ C □ D □ E □ F □

Supponi ora di essere Caio, e **supponi che le scelte di Tizio siano determinate dalle scelte dei partecipanti a questo test nei panni di Tizio**; cosa scegli?

✎ A □ B □ C □ D □ E □ F □

Supponi ancora di essere Caio, e **supponi che le scelte di Tizio siano fatte da un automa che sceglie sempre in modo da vincere il premio**; cosa scegli?

✎ A □ B □ C □ D □ E □ F □

Gioco 4. Tizio e Caio si sfidano in un gioco pericoloso e dalle regole singolari.
Tizio sceglie un'arma, che poi Caio usa per sferrare un colpo a Tizio. **Se Caio colpisce Tizio, allora Caio vince la sfida. Se non lo colpisce, è Tizio che vince.**
Tizio sceglie tra pistola, fioretto, arco e mazza ferrata. Caio prende l'arma scelta da Tizio e sferra un colpo, decidendo se "mirare alla testa" o "mirare al corpo". La probabilità che il colpo vada a segno dipende dall'arma scelta da Tizio e da dove Caio ha mirato, nel seguente modo:
- con la pistola, Caio colpisce al 60% se mira al corpo e al 30% se mira alla testa.
- con il fioretto, Caio colpisce al 50% sia se mira alla testa che al corpo.
- con l'arco, Caio colpisce al 10% se mira al corpo e al 55% se mira alla testa.
- con la mazza ferrata, Caio colpisce al 25% se mira al corpo e al 60% se mira alla testa.

Supponi di essere Tizio, e **supponi che le scelte di Caio siano determinate dalle scelte dei partecipanti a questo test nei panni di Caio**; cosa scegli?

✎ pistola □ fioretto □ arco □ mazza ferrata □

Supponi ora di essere Caio, cosa scegli? [rispondi per tutte le scelte di Tizio]

Se Tizio sceglie pistola, tu scegli di mirare: ✎ alla testa □ al corpo □
Se Tizio sceglie fioretto, tu scegli di mirare: ✎ alla testa □ al corpo □
Se Tizio sceglie arco, tu scegli di mirare: ✎ alla testa □ al corpo □
Se Tizio sceglie mazza ferrata, tu scegli di mirare: ✎ alla testa □ al corpo □

Supponi ancora di essere Tizio, e **supponi che le scelte di Caio siano fatte da un automa che sceglie cercando la massima probabilità della propria vittoria**; cosa scegli?

✎ pistola □ fioretto □ arco □ mazza ferrata □

Gioco 5. Scegli un colore tra i seguenti, con l'**obiettivo di scegliere quello che verrà scelto dal maggior numero di altri partecipanti a questo test**.

✎ marrone □ rosso □ blu □ giallo □ verde □ grigio □

Sempre con l'**obiettivo di scegliere ciò che verrà scelto dal maggior numero degli altri partecipanti a questo test**, scegli un numero tra quelli elencati più sotto dopo aver letto la seguente frase: "Ogni anno è fatto di dodici mesi."

✎ 18 □ 9 □ 24 □ 12 □ 5 □ 31 □

Gioco 6. L'obiettivo di questo gioco è scegliere un numero che si avvicini il più possibile ai due terzi (circa il 66%) della **media dei numeri scelti per questo stesso gioco dai partecipanti al test.** (Ad esempio, se la media dei numeri scelti è 30, tu ottieni il massimo punteggio scegliendo 20, cioè i due terzi di 30). Scegli un numero intero tra 1 e 90: ✎ .....

Ora scegli un numero che si avvicini il più possibile ai due terzi (circa il 66%) della media dei numeri scelti per questo stesso gioco dai partecipanti al test che **hanno risposto correttamente a tutte le domande D4.1, D4.2, D4.3.** Scegli un numero intero tra 1 e 90: ✎ .....

Infine, scegli un numero che si avvicini, per quanto più possibile, ai due terzi (circa il 66%) del numero scelto da **un automa**. L'automa **effettua la sua scelta dopo aver visto il numero da te scelto** e sceglie sempre il numero più vicino ai 2/3 del tuo.
Scegli un numero intero tra 1 e 90: ✎ .....

Gioco 7. Considera una situazione in cui Tizio e Caio devono spartirsi 100 euro, secondo le seguenti regole.

Tizio propone una divisione; poi Caio decide se accettare la divisione oppure rifiutarla. Se la proposta è accettata da Caio, allora diventa effettiva: gli euro vengono divisi come proposto. Se invece la proposta è rifiutata da Caio, allora tutti i soldi vanno perduti, e sia Tizio che Caio ricevono 0 euro.

Supponi di essere Tizio, e **supponi che Caio sia un automa che, data l'offerta ricevuta, sceglie se accettare o rifiutare in base a ciò che gli garantisce il maggior numero di euro.** Devi fare un'offerta: quanti soldi decidi di offrire a Caio come proposta di spartizione?
(Ricorda: il tuo obiettivo è ottenere più euro possibile per te stesso.)
Indica un numero tra 0 e 100: ✎ ….

Supponi di essere Tizio, e **supponi che la scelta di Caio sia determinata dalle scelte dei partecipanti a questo test date nella domanda D3.2.** Quanti soldi decidi di offrire a Caio come proposta di spartizione?
(Ricorda: il tuo obiettivo è ottenere più euro possibile per te stesso.)
Indica un numero tra 0 e 100: ✎ …..

Gioco 8. Supponi di voler commercializzare un test che misuri in maniera attendibile le capacità strategiche degli individui. In particolare, devi scegliere il prezzo per singola somministrazione del test allo scopo di ottenere **il massimo ricavo.** Il tuo ricavo sarà calcolato utilizzando le **risposte effettivamente date alla domanda D5** dai partecipanti a questo test: se scegli un certo prezzo, venderai il test a quel prezzo ma solo a coloro che hanno dichiarato di essere disposti a spendere almeno quel prezzo per il test.

(Poiché sei interessato al massimo ricavo, **non considerare** in alcun modo gli eventuali costi di produzione e/o distribuzione.)

Prezzo per somministrazione: ✎ ………….. euro

Gioco 9. Due eserciti sono schierati, uno di fronte all'altro, comandati rispettivamente da Tizio e Caio.

Sia Tizio che Caio possono scegliere se caricare o ritirarsi. Queste scelte verranno prese in simultanea: ciascuno deciderà senza sapere cosa ha scelto l'altro.

Ciascun esercito può **vincere**, **pareggiare**, **perdere** o **essere annientato** (vincere è preferibile a pareggiare che a sua volta è preferibile a perdere che a sua volta è preferibile ad essere annientato).

Se entrambi gli eserciti vengono fatti caricare, allora si verificherà un massacro, la battaglia terminerà senza vincitori e nessun superstite rimarrà sul campo; in questo caso entrambi gli eserciti **saranno annientati**. Se invece l'esercito avversario si ritira, allora caricandolo si **vincerà** la battaglia. Se l'esercito avversario carica, ritirandosi si **perderà** la battaglia. Se entrambi gli eserciti si ritirano c'è **pareggio**.

Inoltre, l'esercito di Tizio ha alle spalle un fiume, che è attraversabile solamente per mezzo di un ponte. Tizio, prima di cominciare la battaglia, può decidere di abbattere il ponte. In caso il ponte venga abbattuto, la scelta di ritirarsi causerà però la morte per annegamento di tutti i soldati in ritirata, portando quindi all'**annientamento** dell'esercito. Caio non ha un'analoga possibilità di abbattere il ponte, per cui lui potrà sempre ritirarsi senza che il suo esercito venga annientato.

La seguente tabella riassume cosa può accadere:

	**Tizio carica**	**Tizio si ritira**
**Caio carica**	*il ponte è*<br>*intatto:* entrambi annientati<br>*abbattuto:* entrambi annientati	*il ponte è*<br>*intatto:* Tizio perde, Caio vince<br>*abbattuto:* Caio vince, Tizio annientato
**Caio si ritira**	*il ponte è*<br>*intatto:* Tizio vince, Caio perde<br>*abbattuto:* Tizio vince, Caio perde	*il ponte è*<br>*intatto:* entrambi pareggiano<br>*abbattuto:* entrambi pareggiano

Pertanto:

- **Caricare è sempre la scelta migliore se l'altro esercito si ritira.**
- **Se Tizio non abbatte il ponte, per lui ritirarsi è la scelta migliore se Caio carica.**
- **Se Tizio abbatte il ponte, per lui ritirarsi e caricare portano allo stesso risultato (annientamento) se Caio carica.**
- **Per Caio ritirarsi è sempre la scelta migliore, se Tizio carica.**

Si tenga presente che, prima di decidere cosa fare, **Caio osserva se il ponte è stato abbattuto da Tizio**.

Supponi di essere Tizio, e **supponi che le scelte di Caio siano determinate dalle scelte degli altri partecipanti a questo test nei panni di Caio.** Cosa ordini al tuo esercito?
Distruggere il ponte? ✎ Sì □ No □
Poi cosa ordini: caricare o ritirarsi? ✎ Caricare □ Ritirarsi □

Supponi di essere Caio, e **supponi che le scelte di Tizio siano determinate dalle scelte dei partecipanti a questo test nei panni di Tizio.** Cosa ordini al tuo esercito?
Se Tizio ha fatto distruggere il ponte, che fare? ✎ Caricare □ Ritirarsi □
Se Tizio NON ha fatto distruggere il ponte, che fare? ✎ Caricare □ Ritirarsi □

Supponi di nuovo di essere Tizio, e **supponi che le scelte di Caio siano determinate dalle scelte dei partecipanti a questo test nei panni di Caio che hanno risposto correttamente a tutti i quesiti D4.1, D4.2, D4.3.** Cosa ordini al tuo esercito?
Distruggere il ponte? ✎ Sì □ No □
Poi cosa ordini, caricare o ritirarsi? ✎ Caricare □ Ritirarsi □

Gioco 10. Considera una situazione in cui Tizio e Caio partecipano al seguente gioco a stadi:

stadio 1.

**TIZIO** sceglie di:	**terminare** il gioco con: 200€ a Tizio, 0€ a Caio	oppure	**continuare** il gioco e passare allo stadio 2

stadio 2.

**CAIO** sceglie di:	**terminare** il gioco con: 100€ a Tizio, 300€ a Caio	oppure	**continuare** il gioco e passare allo stadio 3

stadio 3.

**TIZIO** sceglie di:	**terminare** il gioco con: 400€ a Tizio, 200€ a Caio	oppure	**continuare** il gioco e passare allo stadio 4

stadio 4.

**CAIO** sceglie di:	**terminare** il gioco con: 300€ a Tizio, 500€ a Caio	oppure	**continuare** il gioco e passare allo stadio 5

stadio 5.

**TIZIO** sceglie di:	**terminare** il gioco con: 600€ a Tizio, 400€ a Caio	oppure	**continuare** il gioco e passare allo stadio 6

stadio 6.

**CAIO** sceglie di:	**terminare** il gioco con: 500€ a Tizio, 700€ a Caio	oppure	**terminare** il gioco con: 600€ a Tizio, 600€ a Caio

Supponi di essere Tizio, e **supponi che le scelte di Caio siano determinate dalle scelte fatte dagli altri giocatori che partecipano a questo test nei panni di Caio.** Supponi di dover fare in anticipo le tue scelte senza sapere cosa farà Caio. **Tieni presente che entrambi i giocatori conoscono queste istruzioni.**
(Ricorda: il tuo obiettivo è ottenere al termine del gioco più euro possibile per te stesso.)
Cosa scegli? ✎ Termina allo stadio 1 □ Termina allo stadio 3 □
Termina allo stadio 5 □ Passa sempre □

Supponi di essere Caio, e **supponi che le scelte di Tizio siano determinate dalle scelte fatte dagli altri giocatori che partecipano a questo test nei panni di Tizio.** Supponi di dover fare in anticipo le tue scelte senza sapere cosa farà Tizio.
(Ricorda: il tuo obiettivo è ottenere al termine del gioco più euro possibile per te stesso.)
Cosa scegli? ✎ Termina allo stadio 2 □ Termina allo stadio 4 □
Termina allo stadio 6 con 500€ a Tizio, 700€ a Caio □
Termina allo stadio 6 con 600€ a Tizio, 600€ a Caio □

Supponi di nuovo di essere Tizio, e **supponi stavolta che le scelte di Caio siano fatte da un automa che vuole ottenere la massima quantità di euro per sé e riesce a prevedere la tua scelta.** (Ricorda: il tuo obiettivo è ottenere al termine del gioco più euro possibile per te stesso.)
Cosa scegli? ✎ Termina allo stadio 1 □ Termina allo stadio 3 □
Termina allo stadio 5 □ Passa sempre □
